# Relative Populations and IR Spectra of Cu_38_ Cluster at Finite Temperature Based on DFT and Statistical Thermodynamics Calculations

**DOI:** 10.3389/fchem.2022.841964

**Published:** 2022-03-01

**Authors:** Carlos Emiliano Buelna-García, Cesar Castillo-Quevedo, Jesus Manuel Quiroz-Castillo, Edgar Paredes-Sotelo, Manuel Cortez-Valadez, Martha Fabiola Martin-del-Campo-Solis, Tzarara López-Luke, Marycarmen Utrilla-Vázquez, Ana Maria Mendoza-Wilson, Peter L. Rodríguez-Kessler, Alejandro Vazquez-Espinal, Sudip Pan, Aned de Leon-Flores, Jhonny Robert Mis-May, Adán R. Rodríguez-Domínguez, Gerardo Martínez-Guajardo, Jose Luis Cabellos

**Affiliations:** ^1^ Departamento de Investigación en Polímeros y Materiales, Universidad de Sonora, Hermosillo, Mexico; ^2^ Organización Científica y Tecnológica del Desierto, Hermosillo, Mexico; ^3^ Departamento de Fundamentos del Conocimiento, Centro Universitario del Norte, Universidad de Guadalajara, Colotlán, Mexico; ^4^ CONACYT-Departamento de Investigación en Física, Universidad de Sonora, Hermosillo, Mexico; ^5^ Instituto de Investigación en Metalurgia y Materiales, Universidad Michoacana de San Nicolás de Hidalgo, Ciudad Universitaria, Morelia, Mexico; ^6^ Universidad Politécnica de Tapachula, Tapachula, Mexico; ^7^ Coordinación de Tecnología de Alimentos de Origen Vegetal, CIAD, A.C., Hermosillo, Mexico; ^8^ Laboratorio de Química Inorgánica y Materiales Moleculares, Facultad de Ingeniería, Universidad Autonoma de Chile, Santiago, Chile; ^9^ Comput. Theor. Chem. Group Departamento de Ciencias Químicas, Facultad de Ciencias Exactas, Universidad Andres Bello, Santiago, Chile; ^10^ Fachbereich Chemie, Philipps-Universität Marburg, Marburg, Germany; ^11^ Departamento de Ciencias Químico Biologicas, Universidad de Sonora, Hermosillo, Mexico; ^12^ Instituto de Física, Universidad Autónoma de San Luis Potosí, San Luis Potosí, Mexico; ^13^ Unidad Académica de Ciencias Químicas, Área de Ciencias de la Salud, Universidad Autónoma de Zacatecas, Zacatecas, Mexico

**Keywords:** nanothermodynamics, IR, DFT, Cu-nanoclusters, genetic-algorithm, relative populations, temperature

## Abstract

The relative populations of Cu_38_ isomers depend to a great extent on the temperature. Density functional theory and nanothermodynamics can be combined to compute the geometrical optimization of isomers and their spectroscopic properties in an approximate manner. In this article, we investigate entropy-driven isomer distributions of Cu_38_ clusters and the effect of temperature on their IR spectra. An extensive, systematic global search is performed on the potential and free energy surfaces of Cu_38_ using a two-stage strategy to identify the lowest-energy structure and its low-energy neighbors. The effects of temperature on the populations and IR spectra are considered via Boltzmann factors. The computed IR spectrum of each isomer is multiplied by its corresponding Boltzmann weight at finite temperature. Then, they are summed together to produce a final temperature-dependent, Boltzmann-weighted spectrum. Our results show that the disordered structure dominates at high temperatures and the overall Boltzmann-weighted spectrum is composed of a mixture of spectra from several individual isomers.

## Introduction

Nanoclusters are of great interest since they allow us to study the transition from free atoms to bulk condensed systems (Wilcoxon and Abrams, 2006) by analyzing the size-dependent evolution of their properties (Ferrando et al., 2008). In particular, noble-metal nanoclusters (NMCs) have attracted attention in many fields of science due to their interesting plasmonic, catalytic (Mathew and Pradeep, 2014; Inwati et al., 2018), and photophysical properties at the nanoscale (Xavier et al., 2012). Specifically, Cu nanoclusters embedded in a dielectric matrix have attracted attention because of their tunable longitudinal surface plasmon resonance characteristics (Inwati et al., 2018). Copper is cheaper than gold and silver and has high photosensitivity, high thermal and electric conductivities, and optical properties (Zhang et al., 2019) that make it a good candidate for nanodevices (Liu et al., 2015) and nanoelectronics development (Jena and Castleman, 2006). Particularly, Cu_38_ has attracted attention because it has “magic number” structures (Tran and Johnston, 2009), which are defined in terms of geometric and energetic factors and related to the closing of electronic shells (Baletto et al., 2004), much like small sodium clusters (de Heer, 1993). The magicity of the Cu_38_ cluster is due only to energetic considerations (Doye and Wales, 1998; Baletto et al., 2004). In contrast, in small, packed barium clusters with magic numbers, stability is dominated by geometric rather than electronic effects (de Heer, 1993). It is believed that magic structures are the global minimum energy structures on the potential energy surface, and thus reflect the molecular properties of the system (Baletto et al., 2004).

From the experimental point of view, the Cu_38_ cluster has been widely studied via photoelectron spectroscopy (PES) in order to extract the electronic gap of the anionic Cu_38_ cluster resulting in a semiconductor with a 0.33 eV electronic gap (Pettiette et al., 1988; Zhang et al., 2019). PES has also been used to study the anionic Cu_38_ cluster, inferring that its putative global minimum should be an oblate structure instead of a highly symmetric structure (Kostko et al., 2005; Zhang et al., 2019), despite computational results for 38-noble metal atom clusters that frequently find a highly symmetric (cuboctahedral) structure (Fujima and Yamaguchi, 1989; Doye and Wales, 1998; Kostko et al., 2005).

From the theoretical point of view, several density functional theory (DFT)-based studies have been carried out, in which, for example, the thermodynamic properties of the Cu_38_ cluster have been reported (Taylor et al., 2008). The transition states and reaction energies of the water gas shift reaction on a Cu_38_ cluster and Cu slab have also been studied via DFT computations (Qi et al., 2020). In particular, the high-symmetry octahedral structure was reported as the lowest energy structure (Takagi et al., 2017) using the PW91 functional (Perdew et al., 1992), a plane-wave basis set, and the pseudopotential approximation (Itoh et al., 2009). The Cu_38_ cluster has also been investigated using a hybrid strategy (Hijazi and Park, 2010); in which the embedded atom potential method followed by DFT computations with the PBE functional and pseudopotential approximation was used. The authors reported a putative global minimum structure with octahedral (OH) symmetry. The second most stable structure was the incomplete-Mackay icosahedron (IMI) located 0.26 eV above the putative global minimum.

There has been some discussion about the lowest energy structure of the Cu_38_ cluster. Searches for the lowest-energy structure that employed many-body potentials identified a cuboctahedral structure (Doye and Wales, 1998; Grigoryan et al., 2005). Several authors used an empirical potential-energy function containing two-body atomic interactions and found that fivefold symmetry appears to be the putative global minimum in the Cu_38_ cluster (Erkoç, 1994; Erkoç and Shaltaf, 1999). In contrast, previous studies reported the cuboctahedron structure as the putative global minimum (Grigoryan et al., 2006) by employing empirical many-body Gupta and Sutton-Chen potentials; some other works also consider the Cu_38_ octahedron cluster to be the putative global minimum (Itoh et al., 2009; Hijazi and Park, 2010; Takagi et al., 2017), but several others found that the Cu_38_ cluster with truncated octahedron geometry is energetically more stable than other configurations (Darby et al., 2002; Itoh et al., 2009; Hijazi and Park, 2010; Núnez and Johnston, 2010; Park and Hijazi, 2012; Fernández et al., 2015; Zhao et al., 2017). Some of us pointed out that the energies computed via different methods such as DFT, second-order Møllet-Plesset approach (MP2), and Coupled cluster with single-double and perturbative triple (CCSDT) excitations yield different energetic ordering results (de la Puente et al., 1997; Buelna-Garcia et al., 2021; Buelna-Garcia et al., 2021; Castillo-Quevedo et al., 2021). In the case of DFT, the functional and basis set employed, the zero-point energy correction (ZPE), the dispersion energy, and other parameters can change the energetic ordering of the low energy structures (Castillo-Quevedo et al., 2021). Moreover, practical molecular systems and materials must be studied at warm temperatures, so their molecular properties at finite temperature are dominated by Boltzmann distributions of isomers (Baletto and Ferrando, 2005; Li and Truhlar, 2014; Buelna-Garcia et al., 2021; Buelna-Garcia et al., 2021; Castillo-Quevedo et al., 2021), and those of the associated materials are statistical averages over the ensemble of conformations (Mendoza-Wilson et al., 2020; Buelna-Garcia et al., 2021; Buelna-Garcia et al., 2021). Total energy computations using DFT methodology are typically carried out at absolute zero temperature, although the thermal properties of the inhomogeneous electron gas in the mid-1960s were studied (Mermin, 1965). Recently, DFT was extended to finite temperature (Pittalis et al., 2011; Gonis and Däne, 2018; Gazquezet al., 2019), but as far as we know, it has not been implemented in any software.

There are cases where the global minimum structure ceases to be the most likely at high temperatures, so other structures prevail. For instance, in small Ag clusters, the temperature leads to the transition from the initial Face-Centered Cubic (FCC) phase to other structures (Redel et al., 2015), thus temperature promotes face changes in materials. Interestingly, the molecular system minimizes the Gibbs free energy at temperatures other than zero and maximizes the entropy (Buelna-Garcia et al., 2021). Although the search for global and local minima is useful in understanding reactivities and catalytic efficiencies, such studies mostly neglect temperature-dependent entropic contributions to free energy when the temperature increases. Taking temperature into account requires dealing with nanothermodynamics (Hill, 1962; Baletto and Ferrando, 2005; Li et al., 2007; Li and Truhlar, 2014; Grigoryan and Springborg, 2019; Buelna-Garcia et al., 2021; Buelna-Garcia et al., 2021). The thermodynamics of clusters have been studied using various tools (Wales, 1996; Li et al., 2007; Li and Truhlar, 2014; Calvo, 2015; Buelna-Garcia et al., 2021) such as in the molecular-dynamics simulations of boron clusters (Martinez-Guajardo et al., 2015) and Cu_38_ clusters (Zhang et al., 2019).

Cluster properties depend heavily on the cluster structure, size, composition, and temperature. Therefore, the first step to understanding their molecular properties is to elucidate the lowest energy structure and its isomers close in energy (Buelna-Garcia et al., 2021; Buelna-Garcia et al., 2021; Baletto and Ferrando, 2005; Darby et al., 2002; Ohno and Maeda, 2006), which is a complex task due to several factors (Buelna-Garcia et al., 2021; Buelna-Garcia et al., 2021). The second step relies on spectroscopy, which gives insight into the structure and has been proposed as a way of detecting structural transformations within clusters. The influence of temperature on Infrared spectroscopy (IR) has been computed before for a variety of clusters (Even et al., 1989; Buelna-Garcia et al., 2021; Buelna-Garcia et al., 2021). The present paper uses the statistical formulation of thermodynamics and nanothermodynamics (Li et al., 2007; Li and Truhlar, 2014; Buelna-Garcia et al., 2021; Buelna-Garcia et al., 2021) to compute the thermodynamic properties of the neutral Cu_38_ cluster, define its putative global minimum at finite temperature, compute the relative populations among the isomers, and the IR spectra as Boltzmann-weighted spectral sums of individual spectra. Our findings show that an amorphous structure strongly dominates the putative global minimum at high temperatures, whereas the truncated octahedron dominates at low temperatures. The remainder of the manuscript is organized as follows: “*Free Energy Surface Exploration Method and Computational Details*” gives the computational details and a brief overview of the theory and algorithms. The results and discussion are presented in “*Results and Discussion*”. This includes the putative global minimum at room temperature, relative populations at temperatures from 20 to 1500 K, and IR spectra as functions of the temperature. Conclusions are given in “*Conclusion*”.

## Free Energy Surface Exploration Method and Computational Details

The putative global minimum is determined by the enthalpy (at zero temperature) or the Gibbs free energy (at temperatures other than zero). A simple analysis of the Gibbs free energy given by ∆G = ∆H − ∆ST leads to the conclusion that the entropy must be maximized in order to minimize the Gibbs free energy (Sutton and Levchenko, 2020; Buelna-Garcia et al., 2021; Buelna-Garcia et al., 2021). From the theoretical point of view, and in order to understand molecular properties at finite temperature, the lowest Gibbs free energy structure (or the structure with the largest entropy), as well as all structures close in energy to the lowest energy structure (or all high-entropy structures close in entropy to the structure with the highest entropy) must be known (Buelna-Garcia et al., 2021; Buelna-Garcia et al., 2021), considering that experiments are performed at finite temperature.

The search for the global minimum in atomic clusters is a complex task due to the number of possible combinations, which grows exponentially with the number of atoms, leading to a combinatorial explosion problem, among others (Buelna-Garcia et al., 2021; Buelna-Garcia et al., 2021). Despite the difficulty of this task, several algorithms have been successfully employed in a targeted way to explore the potential and free energy surfaces. These are coupled to a local optimizer of any electronic structure package. Examples include the Ab initio Random Structure Searching approach (Pickard and Needs, 2011), simulated annealing (Kirkpatrick et al., 1983; Metropolis et al., 1953; Xiang and Gong, 2000; Xiang et al., 2013; Vlachos et al., 1993; Granville et al., 1994), the kick methodology (Pan et al., 2014; Cui et al., 2015; Vargas-Caamal et al., 2016b,a; Cui et al., 2017; Vargas-Caamal et al., 2015; Flórez et al., 2016; Ravell et al., 2018; Hadad et al., 2014; Saunders, 2004, 1987; Grande-Aztatzi et al., 2014; Mendoza-Wilson et al., 2022), and genetic algorithms (Guo et al., 2017; Dong et al., 2018; Mondal et al., 2016; Ravell et al., 2018; Grande-Aztatzi et al., 2014; Rodríguez-Kessler et al., 2017; Alexandrova et al., 2004; Buelna-Garcia et al., 2021). Global minimum structure searches at the DFT level are too computationally expensive to be applied to intermediate and large cluster sizes since, as mentioned earlier, the number of candidates increases exponentially with the number of atoms. In this paper, we use a two-stage procedure to explore the potential energy surface efficiently. In the primary stage, we perform a global search using an empirical methodology. The Gupta interaction potential is used to describe the Cu-Cu interactions with default parameters taken from Refs. (Cleri and Rosato, 1993; Wilson and Johnston, 2002). It is coupled to the basin hopping global optimization algorithm implemented in Python code and part of the GALGOSON global search code (Buelna-Garcia et al., 2021; Buelna-Garcia et al., 2021). In the second stage, all of the lowest energy structures from the primary stage are symmetrized, which is followed by geometry optimization at the DFT level, using the Gaussian-suite code (Frisch et al., 2009). The calculations employ two exchange-correlation functionals, B3PW91 and PBE, and two basis sets, def2SVP and LANL2DZ, with and without considering the D3 version of Grimme’s dispersion corrections (Grimme et al., 2010), as implemented in the Gaussian 09 code (Frisch et al., 2009). Becke’s hybrid three-parameter (Becke 1993, 1988) exchange-correlation functional in combination with the Perdew and Wang GGA functional PW91 (Perdew et al., 1992; Perdew and Wang, 1992) is known as the B3PW91 exchange-correlation functional. The B3PW91 has been employed in other studies of reactivity in copper clusters, where it has provided good performance (Fernández et al., 2015). The PBE exchange-correlation functional (Perdew et al., 1996) has shown good performance with regarding thermochemical properties (del Campo et al., 2012). The LANL2DZ basis set (Dunning and Hay, 1977) has been used in previous computational studies of copper-based molecular properties with very good agreement with experimental values (Legge et al., 2001). In a previous DFT study, the def2-SVP basis set (Weigend and Ahlrichs, 2005) provided good results in the computation of the Cu-metal ligand bond lengths (Niu et al., 2014). The true minimum energy structures are validated via vibrational analysis.

### Thermochemical Properties

All of the thermodynamic properties of an ensemble of molecules can be derived from the molecular partition function (Takadaet al., 2018; Dzib et al., 2019; Buelna-Garcia et al., 2021; Buelna-Garcia et al., 2021). Similarly, the wave function contains all information about a molecular system (Buelna-Garcia et al., 2021). Previous theoretical studies used the partition function to compute thermodynamic properties of Cu_n_ clusters (*n* = 2, 150) as a function of the temperature and showed that the magic number structures are temperature dependent (Li et al., 2007; Grigoryan and Springborg 2019). The thermodynamics of unsupported neutral Al_n_ (2 < *n* < 65) clusters have also been investigated by evaluating vibrational partition functions. They reported that the dominant cluster structure is temperature dependent (Li and Truhlar, 2014). Aditionally, the atomistic thermodynamics framework has been used to predict material behaviors at realistic temperatures (Sutton and Levchenko, 2020). More recently, the partition function was used by some of us to compute the temperature-dependent relative populations and IR spectra of neutral Be_4_B_8_ and anionic Be_6_B_11_ clusters (Buelna-Garcia et al., 2021; Buelna-Garcia et al., 2021), and a similar procedure was employed to compute reaction rate constants in two representative hydrogen abstraction reactions (Dzib et al., 2019). Regarding the temperature-dependent entropic contributions, the [Fe(pmea)(NCS)2] complex was studied by Brehm et al. (Brehm et al., 2006). In this study, the thermodynamic properties are computed using the partition function Q given in Eq. 1 under the rigid rotor, harmonic oscillator, Born-Oppenheimer, ideal gas, and particle-in-a-box approximations.

$$Q\left(T\right)=\sum_{i}g_{i}e^{{-\text{ΔE}}_{i}{\mathrm{/k}}_{B}T}$$ (1)

In Eq. 1, gi is the degeneracy factor, K_B_ is the Boltzmann constant, T is the temperature, and −∆E_i_ is the total energy of a cluster (Buelna-Garcia et al., 2021; Dzib et al., 2019; McQuarrie, 1975). Within Born Oppenheimer and rigid rotor harmonic oscillator approximations, the partition function Q(T) is factorized into electronic, translational, vibrational, and rotational contributions given by Eq. 2

$$q=q_{trans}q_{rot}q_{vib}q_{elec.}$$ (2)

Table 1 shows the contributions of electronic, translational, vibrational, and rotational to the canonical partition function.

**TABLE 1:** Contributions to the partition function.

Contribution	Partition function
Translational	$q_{trans}={\left(\frac{2\pi mk_{B}T}{h^{2}}\right)}^{\frac{3}{2}}\frac{k_{B}T}{P}$
Rotational linear	$q_{rot}^{1}=\frac{T}{\sigma \Theta_{rot}},\Theta_{rot}=|\frac{h^{2}}{2Ik_{B}}$
Rotational nonlinear	qrotnl=π12σ[T32(ΘrotAΘrotBΘrotC)],Θrotj=h22IjkB,j=A,B,C
Vibrational	$q_{vib}^{pol}=\prod_{i=1}^{n_{01}^{\left[a\right]}}\frac{e^{-\Theta_{vib_{i}}/2T}}{1-e^{-\Theta_{vib_{i}}/T}},\Theta_{vib_{i}}=\frac{hv_{i}}{k_{B}}$
Electronic	${\text{q}}_{\text{elec}}=\omega_{0}$

We considered that the energy gap between the first and higher excited states is greater than $k_{B}T$ ; consequently, the electronic partition function $q={\text{q}}_{\text{elec}}$ is given by ${\text{q}}_{\text{elec}}=\omega_{0}$ . $q_{rot}$ , $q_{rot}^{nl}$ , and q=q were used to compute the internal energy (U), and entropy (S) contributions given in Table 2.

**TABLE 2:** Contributions to internal energy and entropy.

	Internal energy	Entropy
Translational	$U_{trans}=\frac{3}{2}RT$	$S_{trans}=R\left(\ln\;q_{trans}+\frac{5}{2}\right)$
Rotational linear	$U_{rot}^{1}=RT$	$S_{rot}^{l}=R\left(\ln\;q_{rot}^{l}+1\right)$
Rotational nonlinear	$U_{rot}^{nl}=\frac{3}{2}RT$	$S_{rot}^{nl}=R\left(\ln\;q_{rot}^{nl}+\frac{3}{2}\right)$
Vibrational	$U_{vib}^{pol}=R\sum_{i}^{n_{0}^{\left[a\right]}}\Theta_{vib_{i}}\left(\frac{1}{2}+\frac{1}{e^{\Theta_{vib_{i}}/T}-1}\right)$	$S_{vib}^{pol}=R\sum_{i}^{n_{o}}\left[\frac{\Theta_{vib_{i}}/T}{e^{\Theta_{vib_{i}}/T}-1}-\ln\left(1\right)-e^{-\Theta_{vib_{i}}/T}\right]$
	$\Theta_{vib_{i}}=\frac{hv_{i}}{k_{B}}$	
Electronic	$U_{elec}=0$	$S_{elec}=R\;\ln\;q_{elec}$

Equations shows in Tables 1 and 2 are the same as those employed in previous studies (Li et al., 2007; Grimme, 2012; Buelna-Garcia et al., 2021; Buelna-Garcia et al., 2021; Dzib et al., 2019) and any standard thermodynamics textbook (McQuarrie, 1975; Hill, 1986).

The vibrational frequencies are computed employing Gaussian code. The Gibbs free energy (G) and the enthalpy (H) are computed employing Eqs. 3 and 4, respectively. In these Equations R is the ideal gas constant, n is the amount of substance, and T is the absoulte temperature.

H=U+nRT. (3)

G=H−TS. (4)

To compute the probability of the occurrence of one particular Cu_38_ cluster in a Boltzmann ensemble at thermal equilibrium as a function of temperature, we employed the probability of occurrence (Shortle, 2003; Li et al., 2007; Grimme, 2012; Bhattacharya et al., 2017; Schebarchov et al., 2018; Dzib et al., 2019; Goldsmith et al., 2019; Grigoryan and Springborg, 2019; Mendoza-Wilson et al., 2020; Bhumla et al., 2021; Buelna-Garcia et al., 2021; Buelna-Garcia et al., 2021) given by Eq. 5:

$$P\left(T\right)=\frac{e^{-\beta \Delta G^{K}}}{\sum e^{-\beta \Delta G^{K}}}.$$ (5)

where $\beta \;=\;1/k_{B}T$ , $k_{B}$ is the Boltzmann constant, T is the temperature, and ΔG k is the Gibbs free energy of the *k*th isomer. We point out that Gibbs free energies must be corrected based on the symmetry. Our previous work showed that the contribution of the rotational entropy to the Gibbs free energy depends on the symmetry, varies linearly with the temperature, and can be significant (Buelna-Garcia et al., 2021). Eq. 5 is restricted so that the sum of all occurrence probabilities at fixed temperature T, i.e., the sum of all Pi (T), is equal to 1. This is given by Eq. 6

$$\sum_{i=1}^{n}P\left(T\right)=1$$ (6)

In this study, the Boltzmann-weighted IR spectrum at a finite temperature is given by Eq. 7:

$$IR=\sum_{i=1}^{n}\left(IR_{i}\right)P_{\left(i\right)}\left(T\right),$$ (7)

where n is the total number of clusters in the ensemble, IR_i_ is the spectrum of the *i*th isomer at temperature T = 0, and Pi (T) is the probability of the *i*th isomer, which is given by Eq. 5. We use the Boltzmann-optics-full-Ader code (BOFA) to compute the occurrence probability and the IR spectra (Buelna-Garcia et al., 2021).

## Results and Discussion

### Low-Energy Structures

**FIGURE 1:**
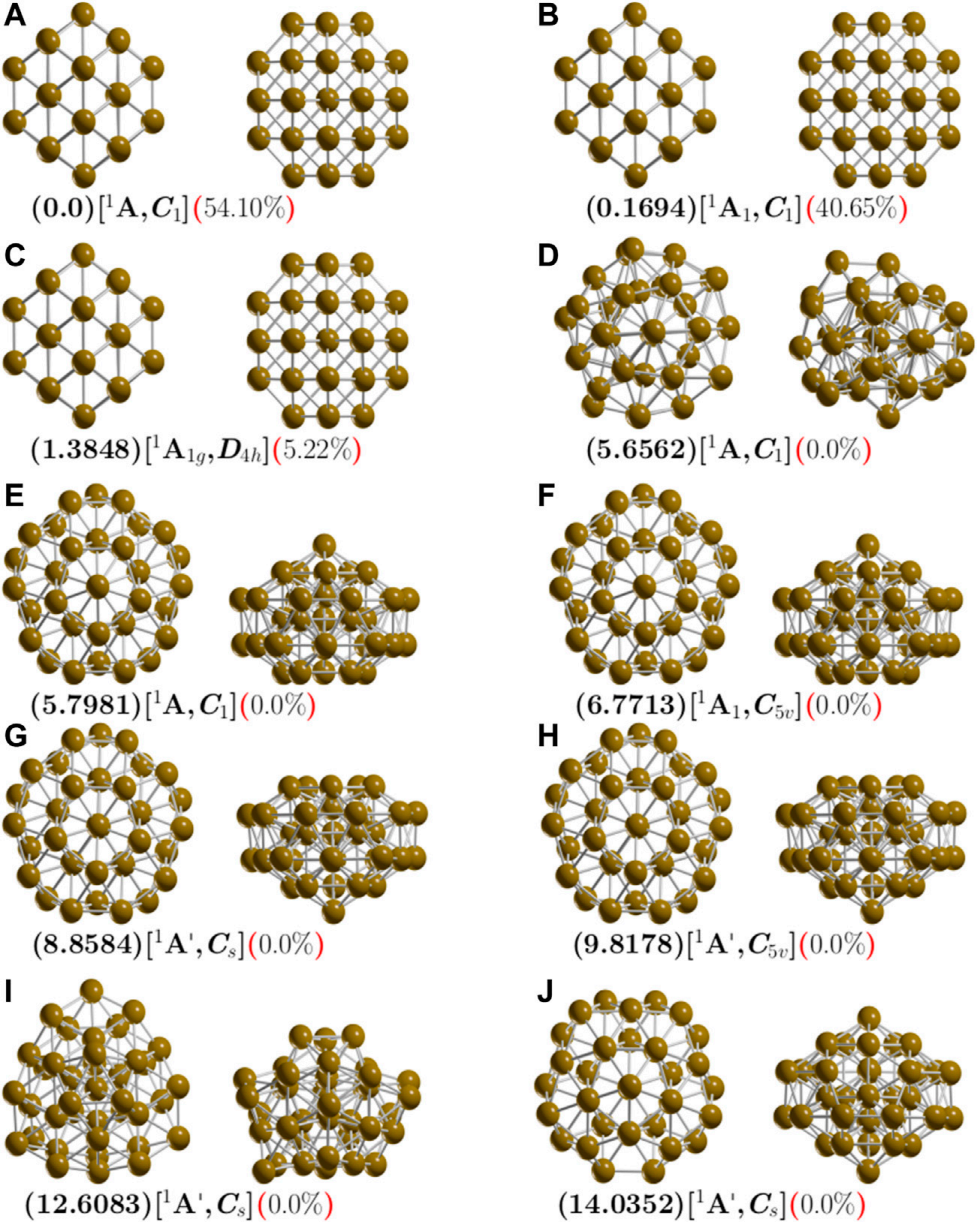
(Color online) Optimized geometries in front and side views of neutral Cu_38_ clusters at the PBE-D3/def2-SVP level of theory. The calculations consider the D3 version of Grimme’s dispersion corrections and the ZPE correction energy. The first letter is the isomer label, relative Gibbs free energies are in kcal/mol (in round parenthesis) at 298.15 K, electronic groups and symmetry point groups are [in square parenthesis], and the probability of occurrence is shown (in round, red parenthesis) at 298.15 K.

The ball and stick models shown in Figure 1 depict the lowest-energy structures of neutral Cu_38_ clusters and some competing isomers. We use the B3PW91/def2SVP level of theory and consider the Grimme (DFT-D3) dispersion pairwise correction (Grimme et al., 2010), at room temperature and at 1 atm pressure. We found that the tetrakaidecahedron is the lowest energy structure. It has fourteen faces: six equivalent square FCC(100) faces and eight equivalent hexagons. This shape is obtained when cutting the corners off a 3D diamond shape. It is an FCC-like truncated octahedron (TO). The calculated structure belongs to the C_1_ symmetry point group and to the ^1^A electronic ground state. Its lowest IR active vibration frequency is 32.57 cm^−1^ and it is a semiconductor with an electronic gap of 0.623 eV. Previous works regarding the exploration of the potential energy surface of Cu_38_ using genetic algorithms via the Gupta potential have often found highly symmetric TO structures (Kostko et al., 2005; Darby et al., 2002), which have also been reported using the Sutton-Chen potential with Monte Carlo simulations (Zhao et al., 2017). The optimized Cu-Cu bond length is 2.4670 Å, which is in good agreement with the reported bond length in the Cu-Cu dimer via DFT calculations (2.248 Å) (Kabir et al., 2004; Guvelioglu et al., 2006) and is consistent with the experimental Cu-Cu bonding distance of 2.22 Å (Kabir et al., 2004). Our computed TO structure diameter is 7.8 Å, which is in good agreement with the 8 Å reported in previous DFT calculations (Guvelioglu et al., 2006).

The structure with the second-lowest energy lies at 0.16 kcal/mol at 298.15 K above the putative global minimum and is also a TO structure with C_1_ symmetry and ^1^A electronic ground state. Its lowest IR active vibration frequency is 32.13 cm^−1^ and it is a semiconductor with an electronic gap of 0.623 eV, thus, it is fairly similar to the putative global minimum. The next structure is slightly higher in energy. It is located 1.38 kcal/mol above the putative global minimum and is also a TO structure. It has D_4h_ symmetry and ^1^A_1g_ electronic ground state. Its lowest IR active vibration frequency is 33.44 cm^−1^. We also explore TO structures, starting with highly-symmetric OH and TH. After geometry optimization without constraints, the OH and TH symmetries become C_1_ and D_4h_. The perfect OH symmetry could be deformed due to the Jahn–Teller effect (Kabir et al., 2004; Guvelioglu et al., 2006). This effect must be considered when calculating the total energy (Zlatar et al., 2010; Zhang et al., 2018) because the computed optical properties could change due to relative population at finite temperatures (Opik and Pryce, 1957). In one of our recent works, we clarified the origin of Gibbs free energy differences between two similar structures with different symmetry point group due to rotational entropy, specifically the RTln(σ) factor (Buelna-García et al., 2021). In this work, the energy difference of 0.16 kcal/mol between the two isomers depicted in Figure 1A with C_1_ symmetries and a root-mean-square deviation (RMSD) of 0.08 is due to the Jahn-Teller effect. The structure located 1.38 kcal/mol above the putative global minimum with D4h symmetry exists due to rotational entropy. The next structure, shown in Figure 1D is located 5.65 kcal/mol above the putative global minimum and has point group symmetry C_1_ and electronic ground state ^1^A. Its lowest IR active vibration frequency is 24.16 cm^−1^. It is a distorted-structure semiconductor with an electronic gap of 1.0 eV, calculated Cu-Cu bond distance of 2.50 Å and molecular diameter of 9.1 Å. Its Cu-Cu bond distance and diameter are slightly larger than the global minimum. This structure possesses the smallest relative ZPE energy, as shown in Supplementary Figure S1, and the smallest vibrational frequency mode of all the isomers. The next two higher energy structures are shown in Figures 1E,F reach 5.8 kcal/mol. They are IMIs with C_1_ and C_5V_ point group symmetries and electronic ground states 1 and ^1^A_1_, respectively. In both cases, the molecular diameter is 8.54 Å, the electronic gap is 0.97 eV, and the Cu-Cu bonding distance is 2.47 Å. Other, higher energy structures are shown in Figures 1G–J. These do not contribute to any molecular properties at zero and finite temperatures. Supplementary Figure S2 depicts lowest-energy-structure screening at the B3PW91/def2SVP level without considering the Grimme D3 atom-pairwise correction. The lowest energy structure is the IMI structure with point group symmetry C_1_ and electronic ground state ^1^A. The molecular diameter is 8.69 Å, which is slightly larger than that of the TO structure (7.8 Å). The average bond distance is 2.50 Å. We find the IMI structure to be the most stable at the PBE-D3/Def2SVP level. In contrast, at the PBE-D3/LANL2DZ level, we find the TO structure to be the putative global minimum. A complete description of the structures located at higher energies is presented in the Supplementary Material. For the Cu_38_ clusters, we point out that the energetic ordering of the isomers, the energy gaps among the isomers, and the putative global minimum interchange when we consider the dispersion interactions.

### Energetics

Employing different methods to compute energies yields different results due to differences in the functional and basis set (Yanai et al., 2004), and the resulting energetic ordering changes (de la Puente et al., 1997; Buelna-Garcia et al., 2021). A comparison of total energy, among isomers, computed with two different exchange-correlation functionals and two basis sets, one which does and one which does not consider the Grimme D3 dispersion, is shown in Table 3. The optimizations performed at the B3PW91/PBE-def2TZVP level that consider the dispersion yield the same type of lowest-energy equilibrium geometries and similar energetic isomer ordering. From the energetic point of view, the inclusion of dispersion is more important than the type of functional or basis set. The first line of Table 3 shows the relative Gibbs free energies computed at the B3PW91-D3/def2TZVP level of theory. The isomer labeled ib in Table 3 and depicted in Figure 1B is located 0.16 kcal/mol above the putative global minimum. In contrast, the second line of Table 3 shows the relative Gibbs free energies computed at the B3PW91/def2TZVP level of theory. Here, the isomer ib in Table 3 that is depicted in Figure 1B is located 0.95 kcal/mol above the putative global minimum. For isomer with label ib, the inclusion of dispersion decreases the Gibbs free energy relative to the putative global minimum (from 0.95 to 0.16 kcal/mol). For isomer with label ic, considering dispersion decreases the Gibbs free energy relative to the putative global minimum from 2.0 to 1.38 kcal/mol. In contrast, the inclusion of dispersion for isomer with label id, the relative Gibbs free energy increases from 2.4 to 5.65 kcal/mol. In summary, considering dispersion reduces the Gibbs free energies of the lowest-energy structures where the Boltzmann factors are not zero. An overall comparison of free energies computed using functional B3PW91, the second line of Table 3, and PBE in four-line in Table 3, shows a reduction in the relative Gibbs free energies when the PBE functional is employed. The LANL2DZ basis set increases the relative Gibbs free energies of the low-energy isomers, as shown by comparing line six and one of Table 3.

**TABLE 3:** A comparison of the energetic isomer ordering as determined using the B3PW91/Def2SVP, PBE/Def2SVP, and PBE/LANL2DZ levels of theory. The Gibbs free energy is computed at room temperature. The electronic energy includes the ZPE energy correction.

Level of theory	Isomers (energy kcal/mol)									
	Energy	i_a_	i_b_	i_c_	i_d_	i_e_	i_f_	i_g_	i_h_	i_i_
B B3PW91-D3/def2SVP	ΔG	0.0	0.16	1.38	5.65	5.79	5.81	6.76	8.85	9.81
	$\varepsilon_{0}+\varepsilon_{ZPE}$	0.	0.09	0.0	5.01	5.01	5.01	5.01	5.01	8.17
	$\varepsilon_{0}$	0.05	0.0	0.10	8.76	4.89	4.89	4.88	4.88	4.88
B B3PW91/def2SVP	ΔG	0.0	0.95	2.0	2.40	2.73	2.91	2.94	3.28	3.32
	$\varepsilon_{0}+\varepsilon_{ZPE}$	0.0	0.0	2.14	3.29	6.52	3.84	2.14	3.27	3.84
	$\varepsilon_{0}$	0.0	0.0	2.09	3.27	6.13	3.76	2.09	3.28	3.76
B PBE-D3/def2SVP	ΔG	0.0	0.86	0.92	5.02	7.23	7.59	7.81	8.88	12.27
	$\varepsilon_{0}+\varepsilon_{ZPE}$	0.0	0.89	0.0	8.70	7.94	7.92	8.61	7.94	14.24
	$\varepsilon_{0}$	0.0	0.90	0.0	9.14	7.92	7.93	8.84	7.94	12.47
P PBE/def2SVP	ΔG	0.0	0.34	0.92	1.37	1.38	1.77	2.82	5.79	8.70
	$\varepsilon_{0}+\varepsilon_{ZPE}$	0.0	0.0	0.37	0.41	1.77	1.77	1.77	9.08	15.75
	$\varepsilon_{0}$	0.0	0.0	0.38	0.39	1.73	1.73	1.74	9.47	9.86
P PBE-D3/LANL2DZ	ΔG	0.0	1.02	3.37	3.46	8.63	9.11	9.59	9.62	9.74
	$\varepsilon_{0}+\varepsilon_{ZPE}$	2.03	0.0	2.12	2.14	8.31	8.77	8.73	9.34	8.71
	$\varepsilon_{0}$	1.97	0.0	2.01	2.01	8.08	8.60	8.52	9.12	8.45
P PBE/LANL2DZP	ΔG	0.0	2.15	2.87	3.03	3.26	4.31	8.89	9.66	9.85
	$\varepsilon_{0}+\varepsilon_{ZPE}$	0.97	0.0	1.46	1.38	1.64	1.68	7.98	9.19	9.58
	$\varepsilon_{0}$	0.86	0.0	1.27	1.11	1.48	1.42	8.03	8.99	9

### Occurrence Probabilities at Finite Temperature

**FIGURE 2:**
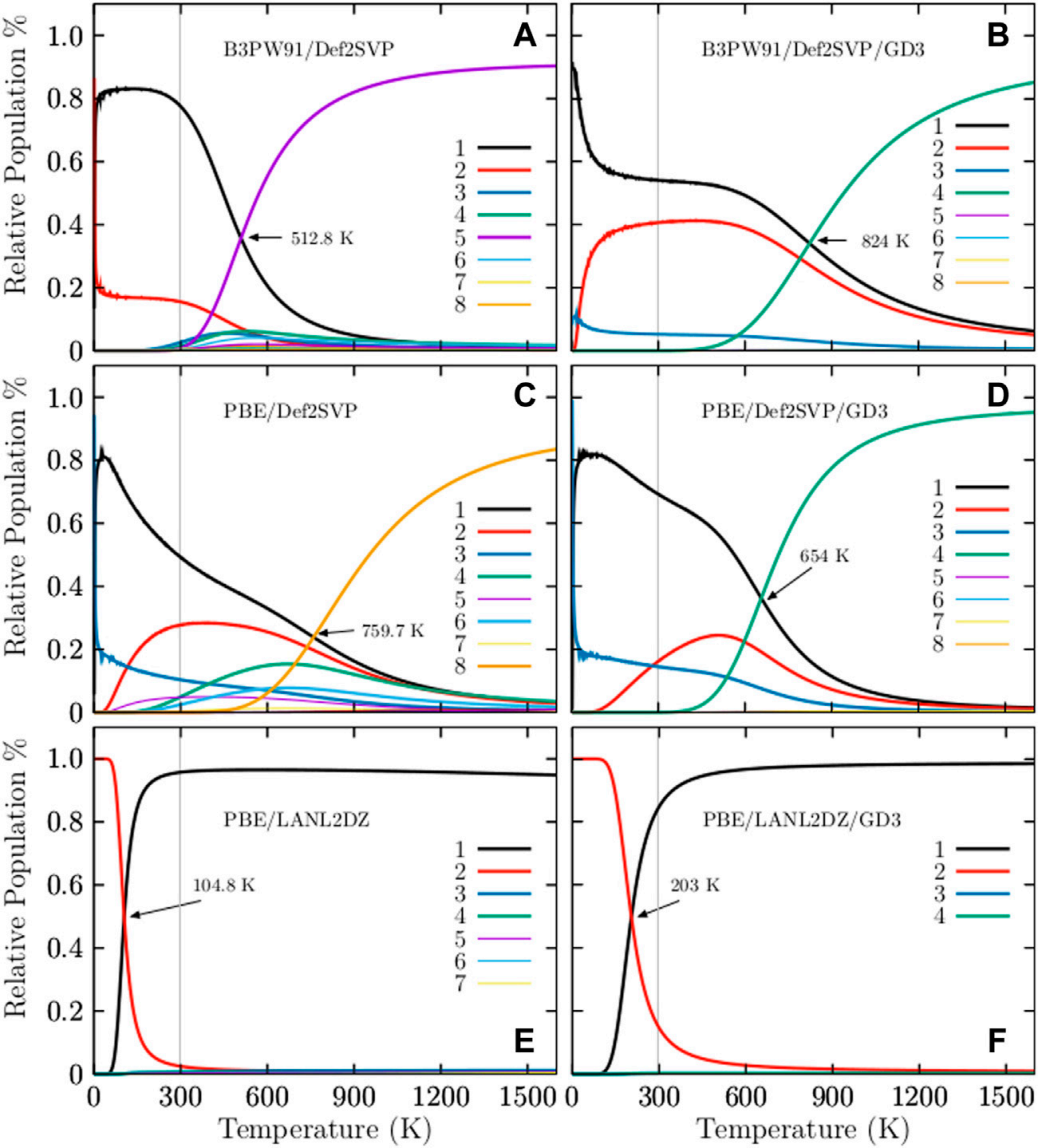
(Color online) The occurrence probabilities for temperatures ranging from 20 to 1500 K at six different levels of theory: **(A)** B3PW91-D3/def2-SVP, **(B)** B3PW91-D3/def2-SVP, **(C)** PBE/def2-SVP, **(D)** PBE-D3/def2-SVP, **(E)** PBE/LANL2DZ, and **(F)** PBE-D3/LANL2DZ. The cases **(B**,**D)**, and **(F)** are computed considering the D3 Grimme dispersion. In all cases, the effect of the dispersion on the solid-solid transformation point in the temperature scale is large for the Cu_38_ cluster. At hot temperatures the dominant structure is an amorphous geometry depicted in Figure 1D, whereas, the TO structure depicted in Figure 1A is the strongly dominant structure at cold temperatures and at the B3PW91-D3/def2-SVP level of theory.

Figure 2 shows the relative populations of neutral Cu_38_ clusters computed at different levels of theory for temperatures ranging from 20 to 1500 K. In order to gain insight into the effects of dispersion on the relative population, this is computed with and without the D3 Grimme dispersion. For ease of comparison, the results are displayed in side-by-side plots in Figure 2.

**FIGURE 3:**
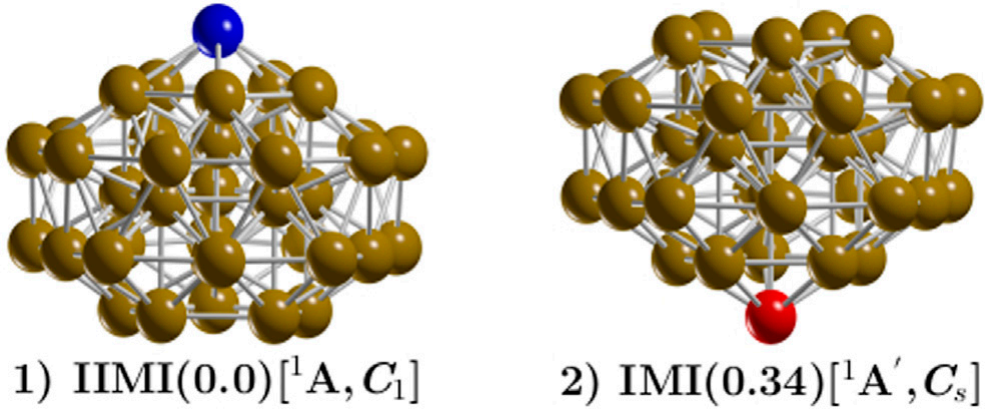
(Color online) The inverted incomplete-Mackay icosahedron (IIMI) is labeled 1 and has C_1_ symmetry. The incomplete-Mackay icosahedron (IMI) is labeled 2, with symmetry C_s_, and is located 0.34 kcal/mol energy above the putative minimum global at 298.15 K. The yellow, red, and blue colored spheres represent copper atoms. The IIMI structure is the result of interchanging the red Cu atom depicted in the IMI structure to the position of the blue atom in the IIMI structure. The IMI structure has been reported in reference Ref Zhang et al. (2019). as the low-energy structure. The Highest Occupied Molecular Orbital (HOMO)—Lowest Unoccupied Molecular Orbital (LUMO) gap of the IMI structure is 0.24 eV (0.356 eV reported in previous DFT studies (Zhang et al., 2019). In contrast, the HOMO-LUMO gap for the IIMI structure is 0.30 eV. This, provides a plausible explanation for the higher energetic stability of why the IIMI structure is energetically more stable.

The occurrence probability of the TO structure with C_1_ symmetry at the B3PW91/def2-SVP level of theory is depicted in the solid, black line in Figure 2A. This occurrence probability of the TO structure strongly dominates from 0 to 300 K; thus, all of the molecular properties in this range of temperature are due only to this structure, and the occurrence probability starts to decay exponentially just before 300 K and nearly disappears at 900 K. The probability of finding the amorphous structure with point group symmetry C_1_ is depicted in the solid, violet line in Figure 2A. This probability starts to grow exponentially and becomes dominant at a temperature between 512.8 and 900 K. The TO and the amorphous structure co-exist at a solid-solid transition temperature of 512.8 K. The effect of dispersion can be seen Figure 2B. The relative population is computed at the B3PW91-D3/def2-SVP level of theory. The dispersion effect is dramatic; the solid-solid transformation point shifts from 512.8 to 824 K, an increase of 160%. In Figure 2B, one can see that the molecular properties below 600 K are due to only the TO structure. The probability of finding the amorphous structure, depicted via the solid, green line in Figure 2B, starts to increase exponentially just before 600 K. The TO and the amorphous structure co-exist at 824 K. The probability of finding the TO structure starts to decay exponentially at 600 K and is still around 20% at 900 K. The occurrence probabilities of various Cu_38_ isomers at the PBE/def2-SVP level of theory are displayed in Figure 2C. The dominant putative global minimum structure at T = 0 is the inverted incomplete-Mackay icosahedron (IIMI) structure depicted in Figure 3A with C_1_ symmetry.

The probability of finding the IIMI structure is shown via the black, solid line in Figure 2C. This probability decays almost linearly until a temperature of 1000 K, where the probability of occurrence almost disappears. At the solid-solid transformation point (759.7 K), the IIMI structure co-exists with an amorphous structure. The probability of finding the amorphous structure starts to increase at 600 K and starts to dominate heavily as the putative global minimum above the solid-solid transformation point. The probability of finding the IMI structure is depicted via the red, solid line in Figure 2C. The probability is maximized (30%) at room temperature. Interestingly, the probabilities of the IIMI and IMI structures do not cross at low temperatures. Previous work reported that the IMI structure can be highly competitive at finite temperature (Zhang et al., 2019). Still, our findings show that the amorphous structure with C_1_ symmetry is quite dominant at high temperatures, whereas the IIMI structure is strongly dominant at low temperatures.

For ease of comparison, Figure 3 displays the IIMI and the IMI structures side by side. The IIMI structure dominates at low temperatures. The dispersion effect shifts the solid-solid transformation point down from 759.7 to 654 K, as shown in Figure 2D. The probability of finding the IIMI structure is depicted via the black, solid line as a function of temperature. The probability decays approximately linearly from 50 to 500 K; after that, it decays exponentially until 900 K, where it disappears. At around 400 K, the probability of finding the amorphous structure (depicted via the green, solid line Figure 2D starts to grow exponentially. At 654 K, it co-exists with the IIMI structure. Above 654 K, the amorphous structure becomes energetically favorable.

### IR Spectra at Finite Temperature

**FIGURE 4:**
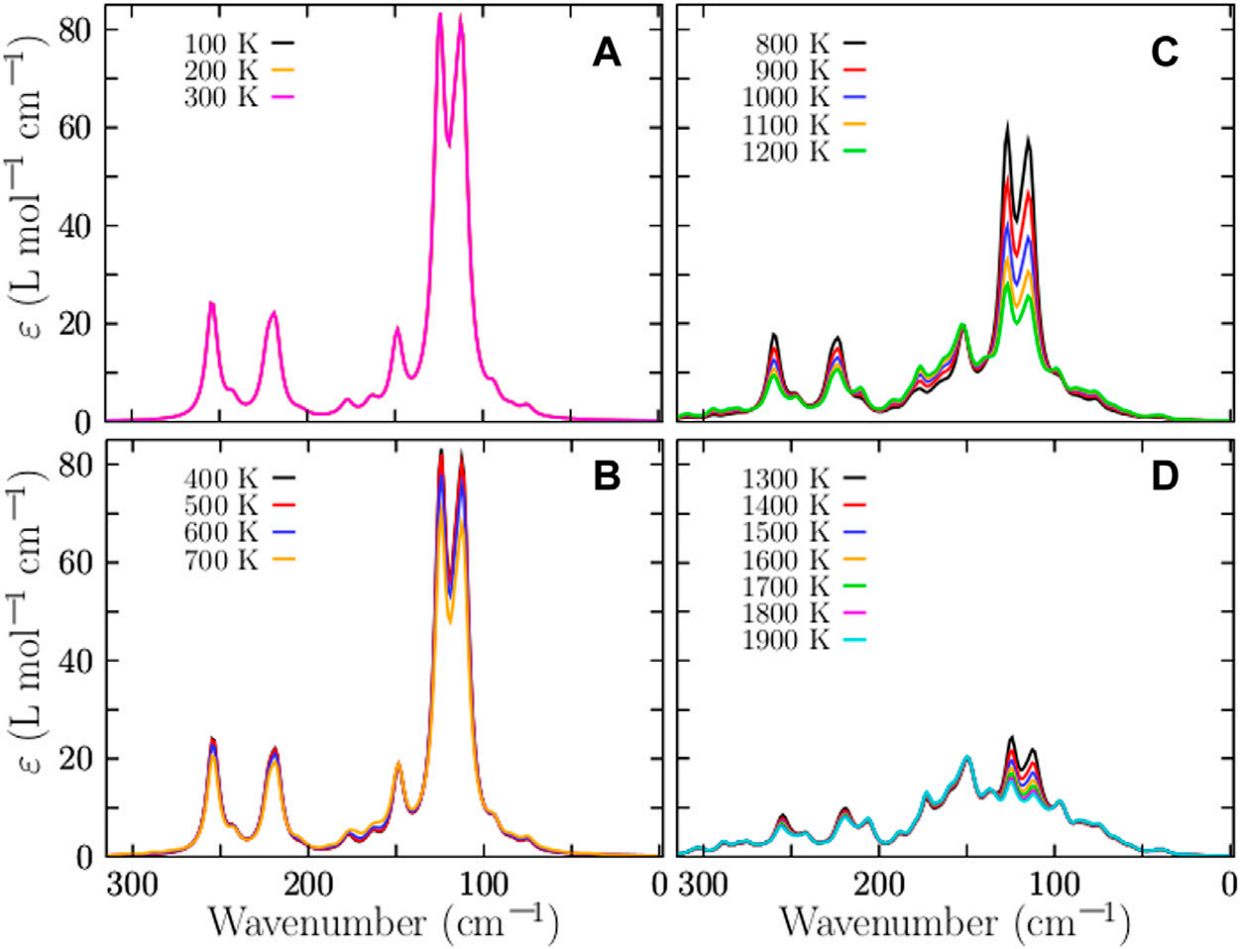
(Color online) Temperature-dependent IR Boltzmann -spectra -weighted at room temperature of the neutral Cu_38_ cluster are shown in panels **(A)**–**(D)** for various temperatures. The computed IR spectrum of each isomer is multiplied by its corresponding Boltzmann weight at finite temperature; Then, they are summed together to produce a final Boltzmann-weighted IR spectrum. Each spectrum of each isomer is computed using density functional theory as implemented in Gaussian code at the B3PW91-D3/def2-TZVP level of theory. The large change in the IR spectra occurs at a temperature of 824 K, as we can see in panel **(D)**, and this agrees with the relative occurrence displayed in Figure 2B. The bulk melting temperature of copper is 1358 K (Grigoryan and Springborg, 2019), considering this, our result below of this temperature are well-behaved.

The properties observed in a molecule are statistical averages over the ensemble of geometrical conformations or isomers accessible to the cluster. Thus, the molecular properties are governed by the Boltzmann distributions of the isomers, which can change significantly with the temperature, primarily due to entropic effects (Buelna-Garcia et al., 2021; Buelna-Garcia et al., 2021; Li et al., 2007). The many soft vibrational modes that the clusters possess are the major contributions to the entropy. The IR spectrum is related to vibrations or rotations that alter the dipole moment and it is observed in molecules with a dipole moment. The IR spectrum is also related to the curvature of the relationship between the potential and the interatomic distance. Complete information regarding molecular vibrations allows us to analyze catalytic chemical reactions (Tinnemans et al., 2006; Brandhorst et al., 2006; Hashimoto et al., 2019). IR spectra are used to identify functional groups and chemical bond information. However, assigning IR bands to vibrational molecular modes in measured spectra can be difficult and requires DFT calculations; as mentioned earlier, the temperature is not considered in these computations and discrepancies between experimental and computed IR spectra can result from finite temperatures, anharmonic effects, and the multi-photon nature of experiments. IR computations assume single-photon processes (Buelna-Garcia et al., 2021). The IR spectra of isolated metal clusters in the gas phase were measured for vanadium cluster cations and neutral and cationic niobium clusters (Fielicke et al., 2005). Even though Cu clusters are important in catalysis and were the first clusters produced experimentally (Powers et al., 1982), the available structural information is limited to photoelectron spectroscopy studies of anions, mass spectrometry, and visible-range photodissociation spectra (Lushchikova et al., 2019). Previous work determined the structures of small cationic copper clusters based on a combination of IR spectroscopy of Cu_n_^+^-Ar_m_ complexes and DFT calculations (Lushchikova et al., 2019). In this work, the IR spectra of the isomers were computed using the Gaussian package under harmonic approximation at the PBPW91-D3 [106]/def2TZVP level and a full width at half maximum of 8 cm^−1^. The Grimme D3 dispersion was considered as implemented in the Gaussian code (Frisch et al., 2009). Imaginary frequencies were checked in all calculations to ensure that the resulting structures were not transition states. The computed frequencies were scaled by a factor of 0.98 to estimate the observed frequencies. Here, the total IR spectrum is computed as a weighted Boltzmann sum of the IR spectrum of each isomer in the distribution at a finite temperature (Buelna-Garcia et al., 2021; Buelna-Garcia et al., 2021; Lecoultre et al., 2011; Sieber et al., 2004). The spectrum is calculated using Eq. 7 and employing the occurrence probabilities displayed in Figure 2. We know of a few theoretical studies on the computation of IR spectra of metal clusters as weighted sums of the IR/UV spectra of the isomers (Sieber et al., 2004). The weighted Boltzmann IR spectra of Cu_38_ clusters at various temperatures are shown in Figure 4. The transition metal clusters are quite stable and their vibrational frequencies are found to be below 400 cm^−1^ (Lapoutre et al., 2013). This is in good agreement with our computed spectra displayed in Figure 4. In particular, the IR spectrum at a low temperature is displayed in Figure 4A. There are three dominant peaks at 125, 225, and 250 cm^−1^. The vibrational mode located at 125 cm^−1^ is a breathing mode that moves the atoms at the surface, whereas the mode at 250 cm^−1^ is a breathing mode where the core atoms move. The IR spectra in Figures 4A,B are similar in the 0–600 K temperature range because the relative populations in this temperature range are dominated strongly by TO structures with C_1_ and D_4h_ structures, as shown in Figure 2B. The IR spectra starts to become small at 700 K. Large changes in the IR spectra happen at a temperature of approximately 800 K, where the solid-solid transition point is located, and where the amorphous structure depicted in Figure 2D and the TO structure depicted in Figure 2A coexist and contribute similarly to the overall IR Boltzmann weighted spectra. Figure 4C shows the IR Boltzmann weighted spectra at 800–1200 K. For temperatures up to 1300 K, the IR Boltzmann weighted spectra are displayed in Figure 4D. At 1200 K, the IR Boltzmann weighted spectra are similar to the spectrum of the amorphous structure depicted in Figure 2D. In contrast, the IR Boltzmann weighted spectra displayed in Figure 4A are similar to the individual IR spectrum of a TO structure. In general, the effect of temperature on the IR spectra is to extenuate the IR spectrum as the temperature increases.

## Conclusion

The temperature and entropic effects produce several competing structures because energy separation between isomers on the free energy surface is small and changes the dominant structure. Thus, it is likely that various isomers interconvert at finite temperature. Our findings show that the amorphous structure with C_1_ symmetry is quite dominant at hot temperatures. These energetically competing structures provide various portions of the overall IR spectrum. In contrast, higher-energy structures with significant energy separation between isomers on the potential/free energy surface do not contribute to the overall IR spectrum. The main contribution to the molecular properties comes from low-energy structures that are close to the global minimum, where the temperature-dependent Boltzmann factor weights are not equal to zero. The Boltzmann-weights depend strongly on the energy separation; the IR spectrum is constant if the energy separation is significant. One motif is dominant in cold conditions and the other in hot conditions. In addition, the level of theory and dispersion influences the location of the T_SS_ point in the temperature scale. Our computations at the six levels of theory clearly show (relative population) that low-symmetry isomers become more stable at high temperatures due to the entropic effect on a Boltzmann ensemble at thermal equilibrium. Our unbiased global search of the free energy surface shows that there is an amorphous structure that dominates at high temperatures. As far as we know, this is a novel high-temperature structure putative global minimum. Computations of the relative populations at a high level of theory is recommended as immediate future work.

## Data Availability

The original contributions presented in the study are included in the article/Supplementary Material, further inquiries can be directed to the corresponding authors.

## References

[B1] Alexandrova A. N. Boldyrev A. I. Fu Y.-J. Yang X. Wang X.-B. Wang L.-S. (2004). Structure of the Na_x_Cl_x+1_ ^−^ (x=1-4) Clusters Viaab Initiogenetic Algorithm and Photoelectron Spectroscopy. J. Chem. Phys. 121, 5709–5719. 10.1063/1.1783276 15366994

[B2] Baletto F. Ferrando R. (2005). Structural Properties of Nanoclusters: Energetic, Thermodynamic, and Kinetic Effects. Rev. Mod. Phys. 77, 371–423. 10.1103/RevModPhys.77.371

[B3] Baletto F. Rapallo A. Rossi G. Ferrando R. (2004). Dynamical Effects in the Formation of Magic Cluster Structures. Phys. Rev. B 69, 235421. 10.1103/PhysRevB.69.235421

[B4] Becke A. D. (1988). Density-functional Exchange-Energy Approximation with Correct Asymptotic Behavior. Phys. Rev. A. 38, 3098–3100. 10.1103/PhysRevA.38.3098 9900728

[B5] Becke A. D. (1993). Density‐functional Thermochemistry. III. The Role of Exact Exchange. J. Chem. Phys. 98, 5648–5652. 10.1063/1.464913

[B6] Bhattacharya S. Berger D. Reuter K. Ghiringhelli L. M. Levchenko S. V. (2017). Theoretical Evidence for Unexpected O-Rich Phases at Corners of MgO Surfaces. Phys. Rev. Mater. 1, 071601. 10.1103/PhysRevMaterials.1.071601

[B7] Bhumla P. Kumar M. Bhattacharya S. (2021). Theoretical Insights into C-H Bond Activation of Methane by Transition Metal Clusters: the Role of Anharmonic Effects. Nanoscale Adv. 3, 575–583. 10.1039/D0NA00669F PMC9417659 36131731

[B8] Brandhorst M. Cristol S. Capron M. Dujardin C. Vezin H. Le bourdon G. (2006). Catalytic Oxidation of Methanol on Mo/Al2O3 Catalyst: An EPR and Raman/infrared Operando Spectroscopies Study. Catal. Today 113, 34–39. 10.1016/j.cattod.2005.11.008 Recent Advances in *In situ* and Operando Studies of Catalytic Reactions

[B9] Brehm G. Reiher M. Le Guennic B. Leibold M. Schindler S. Heinemann F. W. (2006). Investigation of the Low-Spin to High-Spin Transition in a Novel [Fe(pmea)(NCS)2] Complex by IR and Raman Spectroscopy and DFT Calculations. J. Raman Spectrosc. 37, 108–122. 10.1002/jrs.1437

[B10] Buelna-Garcia C. E. Cabellos J. L. Quiroz-Castillo J. M. Martinez-Guajardo G. Castillo-Quevedo C. de Leon-Flores A. (2021). Exploration of Free Energy Surface and thermal Effects on Relative Population and Infrared Spectrum of the Be_6_B_11_ ^−^ Fluxional Cluster. Materials 14. 10.3390/ma14010112 PMC7796227 33383889

[B11] Buelna-García C. E. Robles-Chaparro E. Parra-Arellano T. Quiroz-Castillo J. M. del-Castillo-Castro T. Martínez-Guajardo G. (2021). Theoretical Prediction of Structures, Vibrational Circular Dichroism, and Infrared Spectra of Chiral Be_4_B_8_ Cluster at Different Temperatures. Molecules 26, 3953. 10.3390/molecules26133953 34203563 PMC8271876

[B12] Calvo F. (2015). Thermodynamics of Nanoalloys. Phys. Chem. Chem. Phys. 17, 27922–27939. 10.1039/C5CP00274E 25721192

[B13] Castillo-Quevedo C. Buelna-Garcia C. E. Paredes-Sotelo E. Robles-Chaparro E. Zamora-Gonzalez E. Martin-del-Campo-Solis M. F. (2021). Effects of Temperature on Enantiomerization Energy and Distribution of Isomers in the Chiral Cu_13_ Cluster. Molecules 26, 5710. 10.3390/molecules26185710 34577181 PMC8471510

[B14] Cleri F. Rosato V. (1993). Tight-binding Potentials for Transition Metals and Alloys. Phys. Rev. B 48, 22–33. 10.1103/PhysRevB.48.22 10006745

[B15] Cui Z.-h. Ding Y.-h. Cabellos J. L. Osorio E. Islas R. Restrepo A. (2015). Planar Tetracoordinate Carbons with a Double Bond in CAl3E Clusters. Phys. Chem. Chem. Phys. 17, 8769–8775. 10.1039/C4CP05707D 25739866

[B16] Cui Z.-h. Vassilev-Galindo V. Luis Cabellos J. Osorio E. Orozco M. Pan S. (2017). Planar Pentacoordinate Carbon Atoms Embedded in a Metallocene Framework. Chem. Commun. 53, 138–141. 10.1039/C6CC08273D 27928569

[B17] Darby S. Mortimer-Jones T. V. Johnston R. L. Roberts C. (2002). Theoretical Study of Cu-Au Nanoalloy Clusters Using a Genetic Algorithm. J. Chem. Phys. 116, 1536–1550. 10.1063/1.1429658

[B18] de Heer W. A. (1993). The Physics of Simple Metal Clusters: Experimental Aspects and Simple Models. Rev. Mod. Phys. 65, 611–676. 10.1103/RevModPhys.65.611

[B19] de la Puente E. Aguado A. Ayuela A. López J. M. (1997). Structural and Electronic Properties of Small Neutral (MgO)_n_ Clusers. Phys. Rev. B 56, 7607–7614. 10.1103/PhysRevB.56.7607

[B20] del Campo J. M. Gázquez J. L. Trickey S. B. Vela A. (2012). Non-empirical Improvement of Pbe and its Hybrid PBE0 for General Description of Molecular Properties. J. Chem. Phys. 136, 104108. 10.1063/1.3691197 22423829

[B21] Dong X. Jalife S. Vásquez‐Espinal A. Ravell E. Pan S. Cabellos J. L. (2018). Li_2_B_12_ and Li_3_B_12_: Prediction of the Smallest Tubular and Cage‐like Boron Structures. Angew. Chem. Int. Ed. 57, 4627–4631. 10.1002/anie.201800976 29473272

[B22] Doye J. P. K. Wales D. J. (1998). Global Minima for Transition Metal Clusters Described by Sutton-Chen Potentials. New J. Chem. 22, 733–744. 10.1039/A709249K

[B23] Dunning T. H. Hay P. J. (1977). Gaussian Basis Sets for Molecular Calculations. In Methods of Electronic Structure Theory, ed. Schaefer-H. F. III (Plenum, New York: Springer US), chap. 1. 1-27. 10.1007/978-1-4757-0887-5_1

[B24] Dzib E. Cabellos J. L. Ortíz‐Chi F. Pan S. Galano A. Merino G. (2019). Eyringpy : A Program for Computing Rate Constants in the Gas Phase and in Solution. Int. J. Quan. Chem 119, e25686. 10.1002/qua.25686

[B25] Erkoç S. (1994). An Empirical many-body Potential Energy Function Constructed from Pair-Interactions. Z. Phys. D - Atoms, Mol. Clusters 32, 257–260. 10.1007/BF01437156

[B26] Erkoç Ş. Shaltaf R. (1999). Monte Carlo Computer Simulation of Copper Clusters. Phys. Rev. A. 60, 3053–3057. 10.1103/PhysRevA.60.3053

[B27] Even U. Ben-Horin N. Jortner J. (1989). Multistate Isomerization of Size-Selected Clusters. Phys. Rev. Lett. 62, 140–143. 10.1103/PhysRevLett.62.140 10039933

[B28] Fernández E. Boronat M. Corma A. (2015). Trends in the Reactivity of Molecular O_2_ with Copper Clusters: Influence of Size and Shape. J. Phys. Chem. C 119, 19832–19846. 10.1021/acs.jpcc.5b05023

[B29] Ferrando R. Jellinek J. Johnston R. L. (2008). Nanoalloys: From Theory to Applications of alloy Clusters and Nanoparticles. Chem. Rev. 108, 845–910. 10.1021/cr040090g 18335972

[B30] Fielicke A. von Helden G. Meijer G. (2005). Far-infrared Spectroscopy of Isolated Transition Metal Clusters. Eur. Phys. J. D 34, 83–88. 10.1140/epjd/e2005-00124-7

[B31] Flórez E. Acelas N. Ibargüen C. Mondal S. Cabellos J. L. Merino G. (2016). Microsolvation of NO_3_ ^−^: Structural Exploration and Bonding Analysis. RSC Adv. 6, 71913–71923. 10.1039/C6RA15059D

[B32] Frisch M. J. Trucks G. W. Schlegel H. B. Scuseria G. E. Robb M. A. Cheeseman J. R. (2009). Gaussian 09, Revision B.01. Wallingford: Gaussian, Inc.,.

[B33] Fujima N. Yamaguchi T. (1989). Magnetic Anomaly and Shell Structure of Electronic States of Nickel Microclusters. J. Phys. Soc. Jpn. 58, 3290–3297. 10.1143/JPSJ.58.3290

[B34] Gázquez J. L. Franco‐Pérez M. Ayers P. W. Vela A. (2019). Temperature‐dependent Approach to Chemical Reactivity Concepts in Density Functional Theory. Int. J. Quan. Chem 119, e25797. 10.1002/qua.25797

[B35] Goldsmith B. R. Florian J. Liu J.-X. Gruene P. Lyon J. T. Rayner D. M. (2019). Two-to-three dimensional transition in neutral gold clusters: The crucial role of van der Waals interactions and temperature. Phys. Rev. Mater. 3, 016002. 10.1103/PhysRevMaterials.3.016002

[B36] Gonis A. Däne M. (2018). Extension of the Kohn-Sham Formulation of Density Functional Theory to Finite Temperature. J. Phys. Chem. Sol. 116, 86–99. 10.1016/j.jpcs.2017.12.021

[B37] Grande-Aztatzi R. Martínez-Alanis P. R. Cabellos J. L. Osorio E. Martínez A. Merino G. (2014). Structural Evolution of Small Gold Clusters Doped by One and Two boron Atoms. J. Comput. Chem. 35, 2288–2296. 10.1002/jcc.23748 25284009

[B38] Granville V. Krivanek M. Rasson J.-P. (1994). Simulated Annealing: a Proof of Convergence. IEEE Trans. Pattern Anal. Machine Intell. 16, 652–656. 10.1109/34.295910

[B39] Grigoryan V. G. Alamanova D. Springborg M. (2005). Structure and Energetics of Nickel, Copper, and Gold Clusters. Eur. Phys. J. D 34, 187–190. 10.1140/epjd/e2005-00141-6

[B40] Grigoryan V. G. Alamanova D. Springborg M. (2006). Structure and Energetics of Cu* _N_ * clusters with (2⩽*N*⩽150): An Embedded-Atom-Method Study. Phys. Rev. B 73, 115415. 10.1103/PhysRevB.73.115415

[B41] Grigoryan V. G. Springborg M. (2019). Temperature and Isomeric Effects in Nanoclusters. Phys. Chem. Chem. Phys. 21, 5646–5654. 10.1039/C9CP00123A 30793128

[B42] Grimme S. Antony J. Ehrlich S. Krieg H. (2010). A Consistent and Accurate Ab Initio Parametrization of Density Functional Dispersion Correction (DFT-D) for the 94 Elements H-Pu. J. Chem. Phys. 132, 154104. 10.1063/1.3382344 20423165

[B43] Grimme S. (2012). Supramolecular Binding Thermodynamics by Dispersion-Corrected Density Functional Theory. Chem. Eur. J. 18, 9955–9964. 10.1002/chem.201200497 22782805

[B44] Guo J.-C. Feng L.-Y. Wang Y.-J. Jalife S. Vásquez-Espinal A. Cabellos J. L. (2017). Coaxial Triple-Layered versus Helical Be_6_B_11_ ^−^ Clusters: Dual Structural Fluxionality and Multifold Aromaticity. Angew. Chem. Int. Ed. 56, 10174–10177. 10.1002/anie.201703979 28688126

[B45] Guvelioglu G. H. Ma P. He X. Forrey R. C. Cheng H. (2006). First Principles Studies on the Growth of Small Cu Clusters and the Dissociative Chemisorption ofH2. Phys. Rev. B 73, 155436. 10.1103/PhysRevB.73.155436

[B46] Hadad C. Z. Florez E. Merino G. Cabellos J. L. Ferraro F. Restrepo A. (2014). Potential Energy Surfaces of WC_6_ Clusters in Different Spin States. J. Phys. Chem. A. 118, 5762–5768. 10.1021/jp4099045 24533862

[B47] Hashimoto K. Badarla V. R. Kawai A. Ideguchi T. (2019). Complementary Vibrational Spectroscopy. Nat. Commun. 10, 4411. 10.1038/s41467-019-12442-9 31562337 PMC6764968

[B48] Hijazi I. A. Park Y. H. (2010). Structure of Pure Metallic Nanoclusters: Monte Carlo Simulation and Ab Initio Study. Eur. Phys. J. D 59, 215–221. 10.1140/epjd/e2010-00133-5

[B49] Hill T. (1986). An Introduction to Statistical Thermodynamics. Addison-Wesley series in chemistry. Dover Publications.

[B50] Hill T. L. (1962). Thermodynamics of Small Systems. J. Chem. Phys. 36, 3182–3197. 10.1063/1.1732447

[B51] Inwati G. K. Rao Y. Singh M. (2018). Thermodynamically Induced *In Situ* and Tunable Cu Plasmonic Behaviour. Sci. Rep. 8, 3006. 10.1038/s41598-018-20478-y 29445223 PMC5813046

[B52] Itoh M. Kumar V. Adschiri T. Kawazoe Y. (2009). Comprehensive Study of Sodium, Copper, and Silver Clusters over a Wide Range of Sizes 2≤N≤75. J. Chem. Phys. 131, 174510. 10.1063/1.3187934 19895028

[B53] Jena P. Castleman A. W. (2006). Clusters: A Bridge across the Disciplines of Physics and Chemistry. Proc. Natl. Acad. Sci. 103, 10560–10569. 10.1073/pnas.0601782103 16835306 PMC1636021

[B54] Kabir M. Mookerjee A. Bhattacharya A. K. (2004). Structure and Stability of Copper Clusters: A Tight-Binding Molecular Dynamics Study. Phys. Rev. A. 69, 043203. 10.1103/PhysRevA.69.043203

[B55] Kirkpatrick S. Gelatt C. D. Vecchi M. P. (1983). Optimization by Simulated Annealing. Science 220, 671–680. 10.1126/science.220.4598.671 17813860

[B56] Kostko O. Morgner N. Astruc Hoffmann M. von Issendorff B. (2005). Photoelectron Spectra of Nan- and Cun- with N = 20-40: Observation of Surprising Similarities. Eur. Phys. J. D 34, 133–137. 10.1140/epjd/e2005-00099-3

[B57] Lapoutre V. J. F. Haertelt M. Meijer G. Fielicke A. Bakker J. M. (2013). Communication: Ir Spectroscopy of Neutral Transition Metal Clusters through Thermionic Emission. J. Chem. Phys. 139, 121101. 10.1063/1.4822324 24089710

[B58] Lecoultre S. Rydlo A. Félix C. Buttet J. Gilb S. Harbich W. (2011). Optical Absorption of Small Copper Clusters in Neon: Cun, (N = 1-9). J. Chem. Phys. 134, 074303. 10.1063/1.3552077 21341840

[B59] Legge F. S. Nyberg G. L. Peel J. B. (2001). DFT Calculations for Cu-, Ag-, and Au-Containing Molecules. J. Phys. Chem. A. 105, 7905–7916. 10.1021/jp0101918

[B60] Li Z. H. Jasper A. W. Truhlar D. G. (2007). Structures, Rugged Energetic Landscapes, and Nanothermodynamics of Aln (2 ≤ N ≤ 65) Particles. J. Am. Chem. Soc. 129, 14899–14910. 10.1021/ja073129i 17994736

[B61] Li Z. H. Truhlar D. G. (2014). Nanothermodynamics of Metal Nanoparticles. Chem. Sci. 5, 2605–2624. 10.1039/C4SC00052H

[B62] Liu P. Wang H. Li X. Rui M. Zeng H. (2015). Localized Surface Plasmon Resonance of Cu Nanoparticles by Laser Ablation in Liquid media. RSC Adv. 5, 79738–79745. 10.1039/C5RA14933A

[B63] Lushchikova O. V. Huitema D. M. M. López-Tarifa P. Visscher L. Jamshidi Z. Bakker J. M. (2019). Structures of Cun+ (N = 3-10) Clusters Obtained by Infrared Action Spectroscopy. J. Phys. Chem. Lett. 10, 2151–2155. 10.1021/acs.jpclett.9b00539 30977666 PMC6503464

[B64] Martínez-Guajardo G. Luis Cabellos J. Díaz-Celaya A. Pan S. Islas R. Chattaraj P. K. (2015). Dynamical Behavior of Borospherene: A Nanobubble. Sci. Rep. 5, 11287–11297. 10.1038/srep11287 26096039 PMC4476142

[B65] Mathew A. Pradeep T. (2014). Noble Metal Clusters: Applications in Energy, Environment, and Biology. Part. Part. Syst. Charact. 31, 1017–1053. 10.1002/ppsc.201400033

[B66] McQuarrie D. A. (1975). Statistical Mechanics. Chemistry Series. Harper & Row.

[B67] Mendoza-Wilson A. M. Balandrán-Quintana R. R. Cabellos J. L. (2020). Thermochemical Behavior of Sorghum Procyanidin Trimers with C4-C8 and C4-C6 Interflavan Bonds in the Reaction with Superoxide Anion Radical and H_2_O_2_-Forming NADH-Oxidase Flavoenzyme. Comput. Theor. Chem. 1186, 112912. 10.1016/j.comptc.2020.112912

[B68] Mendoza-Wilson A. M. Balandrán-Quintana R. R. Valdés-Covarrubias M. Á. Cabellos J. L. (2022). Potential of Quercetin in Combination with Antioxidants of Different Polarity Incorporated in Oil-In-Water Nanoemulsions to Control Enzymatic browning of Apples. J. Mol. Struct. 1254, 132372. 10.1016/j.molstruc.2022.132372

[B69] Mermin N. D. (1965). Thermal Properties of the Inhomogeneous Electron Gas. Phys. Rev. 137, A1441–A1443. 10.1103/PhysRev.137.A1441

[B70] Metropolis N. Rosenbluth A. W. Rosenbluth M. N. Teller A. H. Teller E. (1953). Equation of State Calculations by Fast Computing Machines. J. Chem. Phys. 21, 1087–1092. 10.1063/1.1699114 41342507

[B71] Mondal S. Cabellos J. L. Pan S. Osorio E. Torres-Vega J. J. Tiznado W. (2016). 10-π-Electron arenes à la carte: structure and bonding of the [E-(CnHn)-E]n−6 (E = Ca, Sr, Ba; n = 6-8) complexes. Phys. Chem. Chem. Phys. 18, 11909–11918. 10.1039/C6CP00671J 26936126

[B72] Niu S. Huang D.-L. Dau P. D. Liu H.-T. Wang L.-S. Ichiye T. (2014). Assessment of Quantum Mechanical Methods for Copper and Iron Complexes by Photoelectron Spectroscopy. J. Chem. Theor. Comput. 10, 1283–1291. 10.1021/ct400842p PMC3958136 24803858

[B73] Núñez S. Johnston R. L. (2010). Structures and Chemical Ordering of Small Cu−Ag Clusters. J. Phys. Chem. C 114, 13255–13266. 10.1021/jp1048088

[B74] Ohno K. Maeda S. (2006). Global Reaction Route Mapping on Potential Energy Surfaces of Formaldehyde, Formic Acid, and Their Metal-Substituted Analogues. J. Phys. Chem. A. 110, 8933–8941. 10.1021/jp061149l 16836457

[B75] Opik U. Pryce M. H. L. (1957). Studies of the Jahn-Teller Effect. I. A Survey of the Static Problem. Proc. R. Soc. Lond. A. 238, 425–447. 10.1098/rspa.1957.0010

[B76] Pan S. Moreno D. Cabellos J. L. Romero J. Reyes A. Merino G. (2014). In Quest of strong Be-Ng Bonds Among the Neutral Ng-Be Complexes. J. Phys. Chem. A. 118, 487–494. 10.1021/jp409941v 24199587

[B77] Park Y. H. Hijazi I. A. (2012). Critical Size of Transitional Copper Clusters for Ground State Structure Determination: Empirical Andab Initiostudy. Mol. Simulation 38, 241–247. 10.1080/08927022.2011.616502

[B78] Perdew J. P. Burke K. Ernzerhof M. (1996). Generalized Gradient Approximation Made Simple. Phys. Rev. Lett. 77, 3865–3868. 10.1103/PhysRevLett.77.3865 10062328

[B79] Perdew J. P. Chevary J. A. Vosko S. H. Jackson K. A. Pederson M. R. Singh D. J. (1992). Atoms, Molecules, Solids, and Surfaces: Applications of the Generalized Gradient Approximation for Exchange and Correlation. Phys. Rev. B 46, 6671–6687. 10.1103/PhysRevB.46.6671 10002368

[B80] Perdew J. P. Wang Y. (1992). Accurate and Simple Analytic Representation of the Electron-Gas Correlation Energy. Phys. Rev. B 45, 13244–13249. 10.1103/PhysRevB.45.13244 10001404

[B81] Pettiette C. L. Yang S. H. Craycraft M. J. Conceicao J. Laaksonen R. T. Cheshnovsky O. (1988). Ultraviolet Photoelectron Spectroscopy of Copper Clusters. J. Chem. Phys. 88, 5377–5382. 10.1063/1.454575

[B82] Pickard C. J. Needs R. J. (2011). Ab Initiorandom Structure Searching. J. Phys. Condens. Matter 23, 053201. 10.1088/0953-8984/23/5/053201 21406903

[B83] Pittalis S. Proetto C. R. Floris A. Sanna A. Bersier C. Burke K. (2011). Exact Conditions in Finite-Temperature Density-Functional Theory. Phys. Rev. Lett. 107, 163001. 10.1103/physrevlett.107.163001 22107376

[B84] Powers D. E. Hansen S. G. Geusic M. E. Puiu A. C. Hopkins J. B. Dietz T. G. (1982). Supersonic Metal Cluster Beams: Laser Photoionization Studies of Copper Cluster (Cu2). J. Phys. Chem. 86, 2556–2560. 10.1021/j100211a002

[B85] Qi D. Luo X. Yao J. Lu X. Zhang Z. (2020). Computational Study of Reverse Water Gas Shift Reaction on Cu_38_ Cluster Model and Cu Slab Model. J. Theor. Comput. Chem. 19, 2050008. 10.1142/S021963362050008X

[B86] Ravell E. Jalife S. Barroso J. Orozco-Ic M. Hernández-Juárez G. Ortiz-Chi F. (2018). Structure and Bonding in CE5 − (E=Al-Tl) Clusters: Planar Tetracoordinate Carbon versus Pentacoordinate Carbon. Chem. Asian J. 13, 1467–1473. 10.1002/asia.201800261 29575767

[B87] Redel’ L. V. Gafner Y. Y. Gafner S. L. (2015). Role of "magic" Numbers in Structure Formation in Small Silver Nanoclusters. Phys. Solid State. 57, 2117–2125. 10.1134/S106378341510025X

[B88] Rodríguez-Kessler P. L. Pan S. Florez E. Cabellos J. L. Merino G. (2017). Structural Evolution of the Rhodium-Doped Silver Clusters Ag_n_Rh (N ≤ 15) and Their Reactivity toward NO. J. Phys. Chem. C 121, 19420–19427. 10.1021/acs.jpcc.7b05048

[B89] Saunders M. (1987). Stochastic Exploration of Molecular Mechanics Energy Surfaces. Hunting for the Global Minimum. J. Am. Chem. Soc. 109, 3150–3152. 10.1021/ja00244a051

[B90] Saunders M. (2004). Stochastic Search for Isomers on a Quantum Mechanical Surface. J. Comput. Chem. 25, 621–626. 10.1002/jcc.10407 14978704

[B91] Schebarchov D. Baletto F. Wales D. J. (2018). Structure, Thermodynamics, and Rearrangement Mechanisms in Gold Clusters-Insights from the Energy Landscapes Framework. Nanoscale 10, 2004–2016. 10.1039/C7NR07123J 29319705 PMC5901115

[B92] Shortle D. (2003). Propensities, Probabilities, and the Boltzmann Hypothesis. Protein Sci. 12, 1298–1302. 10.1110/ps.0306903 12761401 PMC2323900

[B93] Sieber C. Buttet J. Harbich W. Félix C. Mitrić R. Bonači ć Koutecký V. (2004). Isomer-specific Spectroscopy of Metal Clusters Trapped in a Matrix: Ag_9_ . Phys. Rev. A. 70, 041201. 10.1103/physreva.70.041201

[B94] Sutton C. Levchenko S. V. (2020). First-principles Atomistic Thermodynamics and Configurational Entropy. Front. Chem. 8, 757. 10.3389/fchem.2020.00757 33425844 PMC7793851

[B95] Takada A. Conradt R. Richet P. (2018). Partition Function and Configurational Entropy in Non-equilibrium States: A New Theoretical Model. Entropy 20, 218. 10.3390/e20040218 PMC7512732 33265309

[B96] Takagi N. Ishimura K. Matsui M. Fukuda R. Ehara M. Sakaki S. (2017). Core-Shell versus Other Structures in Binary Cu_38-n_M_n_ Nanoclusters (M = Ru, Rh, Pd, Ag, Os, Ir, Pt, and Au; N = 1, 2, and 6): Theoretical Insight into Determining Factors. J. Phys. Chem. C 121, 10514–10528. 10.1021/acs.jpcc.6b13086

[B97] Taylor C. D. Neurock M. Scully J. R. (2008). First-principles Investigation of the Fundamental Corrosion Properties of a Model Cu[sub 38] Nanoparticle and the (111), (113) Surfaces. J. Electrochem. Soc. 155, C407. 10.1149/1.2926598

[B98] Tinnemans S. J. Mesu J. G. Kervinen K. Visser T. Nijhuis T. A. Beale A. M. (2006). Combining Operando Techniques in One Spectroscopic-Reaction Cell: New Opportunities for Elucidating the Active Site and Related Reaction Mechanism in Catalysis. Catal. Today 113, 3–15. 10.1016/j.cattod.2005.11.076 Recent Advances in *In situ* and Operando Studies of Catalytic Reactions

[B99] Tran D. T. Johnston R. L. (2009). Theoretical Study of Cu_38−n_Au_n_ Clusters Using a Combined Empirical Potential-Density Functional Approach. Phys. Chem. Chem. Phys. 11, 10340–10349. 10.1039/b912501a 19890518

[B100] Vargas-Caamal A. Cabellos J. L. Ortiz-Chi F. Rzepa H. S. Restrepo A. Merino G. (2016a). How many Water Molecules Does it Take to Dissociate HCl? Chem. Eur. J. 22, 2812–2818. 10.1002/chem.201504016 26774026

[B101] Vargas-Caamal A. Ortiz-Chi F. Moreno D. Restrepo A. Merino G. Cabellos J. L. (2015). The Rich and Complex Potential Energy Surface of the Ethanol Dimer. Theor. Chem. Acc. 134, 16. 10.1007/s00214-015-1615-9

[B102] Vargas-Caamal A. Pan S. Ortiz-Chi F. Cabellos J. L. Boto R. A. Contreras-Garcia J. (2016b). How strong Are the Metallocene-Metallocene Interactions? Cases of Ferrocene, Ruthenocene, and Osmocene. Phys. Chem. Chem. Phys. 18, 550–556. 10.1039/C5CP05956A 26618629

[B103] Vlachos D. G. Schmidt L. D. Aris R. (1993). Comparison of Small Metal Clusters: Ni, Pd, Pt, Cu, Ag, Au. Z. Phys. D - Atoms, Mol. Clusters 26, 156–158. 10.1007/BF01425649

[B104] Wales D. J. (1996). Structure, Dynamics, and Thermodynamics of Clusters: Tales from Topographic Potential Surfaces. Science 271, 925–929. 10.1126/science.271.5251.925

[B105] Weigend F. Ahlrichs R. (2005). Balanced Basis Sets of Split Valence, Triple Zeta Valence and Quadruple Zeta Valence Quality for H to Rn: Design and Assessment of Accuracy. Phys. Chem. Chem. Phys. 7, 3297–3305. 10.1039/B508541A 16240044

[B106] Wilcoxon J. P. Abrams B. L. (2006). Synthesis, Structure and Properties of Metal Nanoclusters. Chem. Soc. Rev. 35, 1162–1194. 10.1039/B517312B 17057844

[B107] Wilson N. T. Johnston R. L. (2002). A Theoretical Study of Atom Ordering in Copper-Gold Nanoalloy Clusters. J. Mater. Chem. 12, 2913–2922. 10.1039/B204069G

[B108] Xavier P. L. Chaudhari K. Baksi A. Pradeep T. (2012). Protein-protected Luminescent noble Metal Quantum Clusters: an Emerging Trend in Atomic Cluster Nanoscience. Nano Rev. 3, 14767. 10.3402/nano.v3i0.1476710.3402/nano.v3i0.14767 PMC3272820 22312454

[B109] Xiang Y. Gong X. G. (2000). Efficiency of Generalized Simulated Annealing. Phys. Rev. E 62, 4473–4476. 10.1103/PhysRevE.62.4473 11088992

[B110] Xiang Y. Gubian S. Suomela B. Hoeng J. (2013). Generalized Simulated Annealing for Global Optimization: The GenSA Package. R. J. 5, 13–29. 10.32614/rj-2013-002

[B111] Yanai T. Tew D. P. Handy N. C. (2004). A New Hybrid Exchange-Correlation Functional Using the Coulomb-Attenuating Method (CAM-B3lyp). Chem. Phys. Lett. 393, 51–57. 10.1016/j.cplett.2004.06.011

[B112] Zhang C. Duan H. Lv X. Cao B. Abliz A. Wu Z. (2019). Static and Dynamical Isomerization of Cu_38_ Cluster. Sci. Rep. 9, 7564. 10.1038/s41598-019-44055-z 31110223 PMC6527573

[B113] Zhang J. Wang C.-Z. Zhu Z. Liu Q. H. Ho K.-M. (2018). Multimode Jahn-Teller Effect in Bulk Systems: A Case of the NV0 center in diamond. Phys. Rev. B 97, 165204. 10.1103/physrevb.97.165204

[B114] Zhao B. Zhang R. Huang Z. Wang B. (2017). Effect of the Size of Cu Clusters on Selectivity and Activity of Acetylene Selective Hydrogenation. Appl. Catal. A: Gen. 546, 111–121. 10.1016/j.apcata.2017.08.001

[B115] Zlatar M. Gruden-Pavlović M. Schläpfer C.-W. Daul C. (2010). Intrinsic Distortion Path in the Analysis of the Jahn-Teller Effect. J. Mol. Struct. THEOCHEM 954, 86–93. 10.1016/j.theochem.2010.04.020 DFT 09, 13^th^ International Conference on the Applications of Density Functional Theory in Chemistry and Physics

