# Supplementary material for: Relative Populations and IR Spectra of Cu38 Cluster at Finite Temperature Based on DFT and Statistical Thermodynamics Calculations
Source: Front Chem. 2022 Mar 1;10:841964. doi: 10.3389/fchem.2022.841964 (PMC8921525; doi:10.3389/fchem.2022.841964)
Supplement: Supplementary file 1 [file DataSheet1.doc]

Relative Populations and IR Spectra of Cu_38_ Cluster at Finite Temperature Based on DFT and Statistical Thermodynamics Calculations

**Supplementary Material**

Carlos Emiliano Buelna-García^1,2^, Cesar Castillo-Quevedo^3^, Jesus Quiroz-Castillo^1^ , Edgar Paredes-Sotelo^1^, Manuel Cortez-Valadez^4^, Martha Fabiola Martin-del-Campo-Solis^3^, Tzarara López-Luke^5^, Marycarmen Utrilla-Vázquez^6^, Ana Maria Mendoza-Wilson^7^, Peter L. Rodríguez-Kessler^8^, Alejandro Vazquez-Espinal^9^, Sudip Pan^10^, Aned de Leon-Flores^11^, Jhonny Robert Mis-May^6^, Adán R. Rodríguez-Domínguez^11^, Gerardo Martínez-Guajardo^12*^ and Jose Luis Cabellos ^6*^

^1^Departamento de Investigación en Polímeros y Materiales, Universidad de Sonora, Hermosillo, Mexico, ^2^Organización Científica y Tecnológica del Desierto, Hermosillo, Mexico, ^3^Departamento de Fundamentos del Conocimiento, Centro Universitario del Norte, Universidad de Guadalajara, Colotlán, Mexico, ^4^ CONACYT-Departamento de Investigación en Física, Universidad de Sonora, Hermosillo, Mexico, ^5^Instituto de Investigación en Metalurgia y Materiales, Universidad Michoacana de San Nicolás de Hidalgo, Ciudad Universitaria, Morelia, Mexico ^6^Universidad Politécnica de Tapachula, Tapachula, Mexico, ^7^Coordinación de Tecnología de Alimentos de Origen Vegetal, CIAD, A.C., Hermosillo, Mexico, ^8^Laboratorio de Química Inorgánica y Materiales Moleculares, Facultad de Ingeniería, Universidad Autonoma de Chile, Santiago, Chile, ^9^Comput. Theor. Chem. Group Departamento de Ciencias Químicas, Facultad de Ciencias Exactas, Universidad Andres Bello, Santiago, Chile, ^10^Fachbereich Chemie, Philipps-Universität Marburg, Marburg, Germany, ^11^Departamento de Ciencias Químico Biologicas, Universidad de Sonora, Hermosillo,Mexico, ^12^Instituto de Física, Universidad Autónoma de San Luis Potosí, San Luis Potosí, Mexico, ^13^Unidad Académica deCiencias Químicas, Área de Ciencias de la Salud, Universidad Autónoma de Zacatecas, Zacatecas, Mexico

**Correspondence:**Corresponding Author: Gerardo Martinez-Guajardo, Jose Luis Cabellos
germtzguajardo@uaz.edu.mx, jose.cabellos@unison.mx

Keywords: nanothermodynamic, IR, DFT, Cu_38-_nanoclusters, genetic-algorithm, relative populations, temperature.

1 SUPPLEMENTARY FIGURES


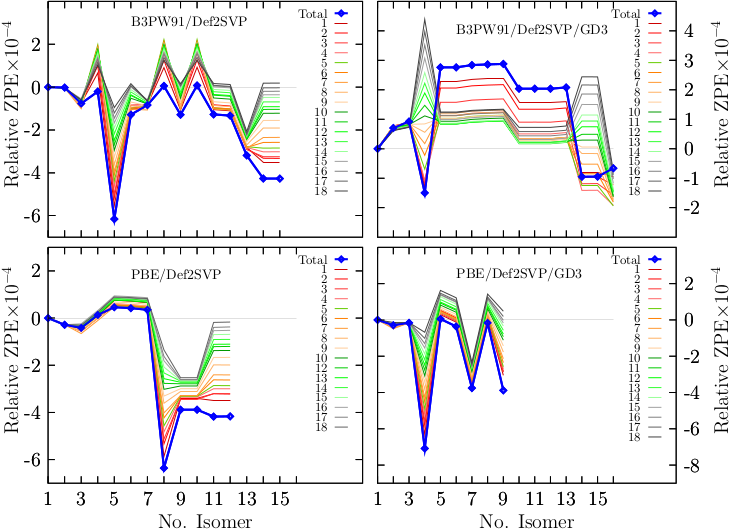


Figure S1 (Color online) Relative zero-point energy (ZPE) decomposition as a function of the number of isomers and vibrational modes, with the reference ZPE of the lowest-energy isomer. The x-axis is the number of isomers. The lowest value of the total relative ZPE as is correlated with the isomer that dominates at hot temperatures, which is the amorphous structure. The blue line depicted the total ZPE, taking all 108 vibrational modes of the cluster.


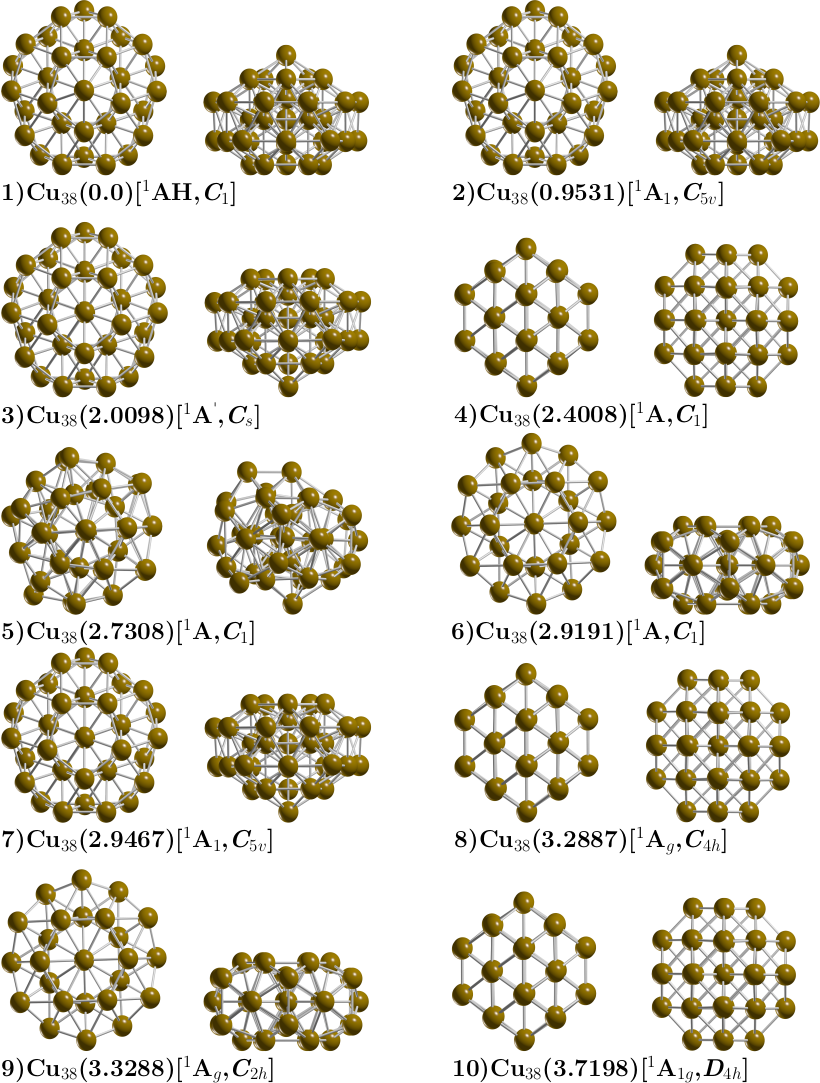


Figure S2 (Color online) Optimized geometries in front and side views of neutral Cu_38_ clusters at the PBE/def2-SVP level of theory. The calculatios take into account the ZPE correction energy. The first letter is the isomer label, relative Gibbs free energies are in kcal/mol (in round parenthesis) at 298.15 K, electronic groups and symmetry point groups are [in square parenthesis].

Atomic coordinates XYZ

38

0.000000000 0001.out

Cu -1.741167000000 -0.158313000000 3.424442000000

Cu -1.777542000000 -0.161558000000 -0.000026000000

Cu -0.158340000000 1.741115000000 3.424465000000

Cu 0.158346000000 -1.741130000000 -3.424473000000

Cu -1.638340000000 -1.965976000000 -1.760408000000

Cu -1.965973000000 1.638364000000 -1.760399000000

Cu -0.161559000000 1.777542000000 0.000001000000

Cu 1.741184000000 0.158312000000 -3.424443000000

Cu -1.638381000000 -1.965997000000 1.760353000000

Cu 1.965966000000 -1.638365000000 1.760395000000

Cu -0.000022000000 -0.000001000000 1.661034000000

Cu 0.158252000000 -1.741146000000 3.424462000000

Cu 2.092781000000 -3.341499000000 0.000013000000

Cu 3.341505000000 2.092789000000 0.000059000000

Cu 1.777542000000 0.161558000000 0.000020000000

Cu 0.317743000000 -3.496543000000 1.724356000000

Cu 0.317778000000 -3.496524000000 -1.724370000000

Cu -3.496557000000 -0.317767000000 1.724319000000

Cu 3.496520000000 0.317741000000 1.724395000000

Cu -1.966017000000 1.638350000000 1.760362000000

Cu 3.664053000000 -1.455978000000 0.000035000000

Cu 0.161557000000 -1.777536000000 -0.000006000000

Cu -3.341499000000 -2.092782000000 -0.000042000000

Cu -3.664057000000 1.455992000000 -0.000039000000

Cu 3.496552000000 0.317775000000 -1.724315000000

Cu 1.638379000000 1.966002000000 -1.760352000000

Cu 1.741096000000 0.158279000000 3.424485000000

Cu -0.317788000000 3.496524000000 1.724365000000

Cu -1.741081000000 -0.158288000000 -3.424488000000

Cu 1.966013000000 -1.638353000000 -1.760365000000

Cu -0.158253000000 1.741137000000 -3.424459000000

Cu -0.317738000000 3.496539000000 -1.724349000000

Cu -1.455990000000 -3.664048000000 -0.000025000000

Cu 0.000024000000 0.000009000000 -1.661040000000

Cu -2.092798000000 3.341499000000 -0.000013000000

Cu 1.455986000000 3.664055000000 0.000042000000

Cu -3.496517000000 -0.317760000000 -1.724399000000

Cu 1.638342000000 1.965984000000 1.760408000000

38

0.169425003 0002.out

Cu 3.513500000000 1.796474000000 0.000001000000

Cu 0.001079000000 1.772700000000 -0.000004000000

Cu 3.516966000000 -0.002124000000 -1.698724000000

Cu -3.516961000000 0.002132000000 1.698727000000

Cu -1.808652000000 1.810631000000 1.758032000000

Cu -1.808655000000 1.810629000000 -1.758028000000

Cu 0.000024000000 -0.000012000000 -1.680316000000

Cu -3.513525000000 -1.796471000000 -0.000010000000

Cu 1.810831000000 1.808461000000 1.758030000000

Cu 1.808648000000 -1.810647000000 1.758046000000

Cu 1.771217000000 -0.001082000000 0.000009000000

Cu 3.516981000000 -0.002120000000 1.698709000000

Cu -0.001045000000 -1.735089000000 3.435940000000

Cu -0.002123000000 -3.498863000000 -1.735691000000

Cu -0.001088000000 -1.772720000000 0.000003000000

Cu 1.769137000000 -0.001066000000 3.418064000000

Cu -1.769051000000 0.001079000000 3.418037000000

Cu 1.765981000000 3.525659000000 -0.000002000000

Cu 1.761732000000 -3.527775000000 0.000020000000

Cu 1.810839000000 1.808464000000 -1.758034000000

Cu -0.002148000000 -3.498870000000 1.735669000000

Cu 0.000016000000 -0.000009000000 1.680314000000

Cu 0.002097000000 3.498861000000 1.735676000000

Cu 0.002097000000 3.498863000000 -1.735698000000

Cu -1.766014000000 -3.525655000000 -0.000018000000

Cu -1.810842000000 -1.808450000000 -1.758047000000

Cu 3.511350000000 -1.800707000000 0.000022000000

Cu 1.769120000000 -0.001061000000 -3.418053000000

Cu -3.511372000000 1.800704000000 0.000010000000

Cu -1.810841000000 -1.808447000000 1.758028000000

Cu -3.516989000000 0.002137000000 -1.698717000000

Cu -1.769072000000 0.001075000000 -3.418030000000

Cu 0.001057000000 1.735091000000 3.435939000000

Cu -1.771212000000 0.001074000000 -0.000010000000

Cu 0.001046000000 1.735085000000 -3.435936000000

Cu -0.001046000000 -1.735083000000 -3.435939000000

Cu -1.761741000000 3.527776000000 0.000002000000

Cu 1.808658000000 -1.810644000000 -1.758021000000

38

1.384892499 0003.out

Cu -3.517960000000 1.780496000000 0.000000000000

Cu 0.000000000000 1.785134000000 0.000000000000

Cu -3.510601000000 0.000000000000 1.725845000000

Cu 3.510601000000 0.000000000000 -1.725845000000

Cu 1.809494000000 1.809494000000 -1.759436000000

Cu 1.809494000000 1.809494000000 1.759436000000

Cu 0.000000000000 0.000000000000 1.661000000000

Cu 3.517960000000 -1.780496000000 0.000000000000

Cu -1.809494000000 1.809494000000 -1.759436000000

Cu -1.809494000000 -1.809494000000 -1.759436000000

Cu -1.785134000000 0.000000000000 0.000000000000

Cu -3.510601000000 0.000000000000 -1.725845000000

Cu 0.000000000000 -1.748410000000 -3.423917000000

Cu 0.000000000000 -3.510601000000 1.725845000000

Cu 0.000000000000 -1.785134000000 0.000000000000

Cu -1.748410000000 0.000000000000 -3.423917000000

Cu 1.748410000000 0.000000000000 -3.423917000000

Cu -1.780496000000 3.517960000000 0.000000000000

Cu -1.780496000000 -3.517960000000 0.000000000000

Cu -1.809494000000 1.809494000000 1.759436000000

Cu 0.000000000000 -3.510601000000 -1.725845000000

Cu 0.000000000000 0.000000000000 -1.661000000000

Cu 0.000000000000 3.510601000000 -1.725845000000

Cu 0.000000000000 3.510601000000 1.725845000000

Cu 1.780496000000 -3.517960000000 0.000000000000

Cu 1.809494000000 -1.809494000000 1.759436000000

Cu -3.517960000000 -1.780496000000 0.000000000000

Cu -1.748410000000 0.000000000000 3.423917000000

Cu 3.517960000000 1.780496000000 0.000000000000

Cu 1.809494000000 -1.809494000000 -1.759436000000

Cu 3.510601000000 0.000000000000 1.725845000000

Cu 1.748410000000 0.000000000000 3.423917000000

Cu 0.000000000000 1.748410000000 -3.423917000000

Cu 1.785134000000 0.000000000000 0.000000000000

Cu 0.000000000000 1.748410000000 3.423917000000

Cu 0.000000000000 -1.748410000000 3.423917000000

Cu 1.780496000000 3.517960000000 0.000000000000

Cu -1.809494000000 -1.809494000000 1.759436000000

38

5.798100000 0006.out

Cu 1.172939000000 1.097826000000 3.378465000000

Cu -0.032807000000 -2.145198000000 0.000000000000

Cu 2.431734000000 -0.653745000000 2.012704000000

Cu -0.938438000000 -2.463862000000 -3.334080000000

Cu -1.123725000000 -0.000130000000 0.000000000000

Cu 1.173103000000 -2.873666000000 -2.087959000000

Cu 1.172939000000 1.097826000000 -3.378465000000

Cu -0.938631000000 2.409535000000 -3.373250000000

Cu -0.032655000000 -0.662804000000 2.040109000000

Cu -0.032401000000 1.735377000000 1.260561000000

Cu 2.431564000000 1.712147000000 1.243736000000

Cu -0.938609000000 0.033485000000 4.145587000000

Cu -2.182325000000 0.675722000000 2.079899000000

Cu -0.938756000000 3.952969000000 1.249204000000

Cu -0.938756000000 3.952969000000 -1.249204000000

Cu 1.145976000000 -1.308226000000 4.026584000000

Cu -2.182373000000 -1.769544000000 1.285501000000

Cu 1.146150000000 -4.233754000000 0.000000000000

Cu 1.145845000000 3.425285000000 2.488512000000

Cu 1.173103000000 -2.873666000000 2.087959000000

Cu -2.182362000000 2.186891000000 0.000000000000

Cu -3.439847000000 -0.000200000000 0.000000000000

Cu -0.938319000000 -3.932561000000 1.312904000000

Cu 2.431736000000 -2.116158000000 0.000000000000

Cu 1.145845000000 3.425285000000 -2.488512000000

Cu -0.032401000000 1.735377000000 -1.260561000000

Cu -0.938631000000 2.409535000000 3.373250000000

Cu 1.270655000000 -0.000203000000 0.000000000000

Cu 1.145976000000 -1.308226000000 -4.026584000000

Cu -2.182373000000 -1.769544000000 -1.285501000000

Cu -0.938609000000 0.033485000000 -4.145587000000

Cu -0.032655000000 -0.662804000000 -2.040109000000

Cu -0.938438000000 -2.463862000000 3.334080000000

Cu -2.182325000000 0.675722000000 -2.079899000000

Cu 2.431734000000 -0.653745000000 -2.012704000000

Cu 1.172889000000 3.552872000000 0.000000000000

Cu -0.938319000000 -3.932561000000 -1.312904000000

Cu 2.431564000000 1.712147000000 -1.243736000000

38

5.798100000 0005.out

Cu 1.105608000000 3.375910000000 -1.172987000000

Cu -2.145190000000 0.004974000000 0.032902000000

Cu -0.649183000000 2.014217000000 -2.431704000000

Cu -2.471547000000 -3.328357000000 0.938545000000

Cu -0.000079000000 -0.000001000000 1.123724000000

Cu -2.878554000000 -2.081286000000 -1.172976000000

Cu 1.089932000000 -3.381006000000 -1.172979000000

Cu 2.401746000000 -3.378831000000 0.938519000000

Cu -0.658068000000 2.041641000000 0.032685000000

Cu 1.738298000000 1.256532000000 0.032323000000

Cu 1.714919000000 1.239763000000 -2.431641000000

Cu 0.043144000000 4.145497000000 0.938610000000

Cu 0.680643000000 2.078324000000 2.182295000000

Cu 3.955899000000 1.240030000000 0.938578000000

Cu 3.950099000000 -1.258371000000 0.938578000000

Cu -1.298933000000 4.029609000000 -1.145915000000

Cu -1.766459000000 1.289600000000 2.182453000000

Cu -4.233794000000 0.009824000000 -1.145961000000

Cu 3.430997000000 2.480561000000 -1.145998000000

Cu -2.868866000000 2.094620000000 -1.172981000000

Cu 2.186984000000 -0.005073000000 2.182263000000

Cu -0.000045000000 0.000000000000 3.439846000000

Cu -3.929462000000 1.322022000000 0.938496000000

Cu -2.116261000000 0.004912000000 -2.431642000000

Cu 3.419450000000 -2.496451000000 -1.145994000000

Cu 1.732447000000 -1.264583000000 0.032321000000

Cu 2.417399000000 3.367646000000 0.938517000000

Cu -0.000260000000 0.000001000000 -1.270656000000

Cu -1.317613000000 -4.023537000000 -1.145918000000

Cu -1.772420000000 -1.281396000000 2.182451000000

Cu 0.023909000000 -4.145655000000 0.938616000000

Cu -0.667531000000 -2.038564000000 0.032684000000

Cu -2.456078000000 3.339789000000 0.938548000000

Cu 0.670994000000 -2.081457000000 2.182292000000

Cu -0.658520000000 -2.011176000000 -2.431704000000

Cu 3.552810000000 -0.008241000000 -1.173050000000

Cu -3.935562000000 -1.303783000000 0.938496000000

Cu 1.709147000000 -1.247704000000 -2.431634000000

38

5.810022499 0007.out

Cu 1.766398000000 -3.082086000000 -1.172799000000

Cu 1.440661000000 1.589530000000 0.032911000000

Cu 1.930492000000 -0.866814000000 -2.431711000000

Cu -0.816402000000 4.064729000000 0.938172000000

Cu -0.000031000000 0.000142000000 1.123925000000

Cu 0.382489000000 3.531342000000 -1.172949000000

Cu -3.240376000000 1.454978000000 -1.172933000000

Cu -4.118083000000 0.479809000000 0.938149000000

Cu 1.956875000000 -0.878715000000 0.032801000000

Cu -0.231030000000 -2.132524000000 0.032845000000

Cu -0.227732000000 -2.103731000000 -2.431572000000

Cu 3.049608000000 -2.808643000000 0.938449000000

Cu 1.087887000000 -1.897327000000 2.182398000000

Cu -1.728917000000 -3.768022000000 0.938368000000

Cu -3.580249000000 -2.090260000000 0.938549000000

Cu 3.862306000000 -1.734288000000 -1.146249000000

Cu 2.140799000000 0.448442000000 2.182400000000

Cu 2.842814000000 3.137229000000 -1.146425000000

Cu -0.455870000000 -4.208980000000 -1.146341000000

Cu 3.477118000000 0.727378000000 -1.172831000000

Cu -1.468555000000 -1.620845000000 2.182378000000

Cu -0.000120000000 0.000172000000 3.440164000000

Cu 3.613691000000 2.032444000000 0.938308000000

Cu 1.420985000000 1.567972000000 -2.431672000000

Cu -4.143913000000 -0.867104000000 -1.146419000000

Cu -2.099748000000 -0.439303000000 0.032870000000

Cu 0.881639000000 -4.050719000000 0.938679000000

Cu -0.000046000000 -0.000099000000 -1.269991000000

Cu -2.105204000000 3.672841000000 -1.146580000000

Cu 0.235173000000 2.174649000000 2.182401000000

Cu -3.094383000000 2.758837000000 0.938321000000

Cu -1.066643000000 1.861156000000 0.032997000000

Cu 4.125010000000 -0.413264000000 0.938640000000

Cu -1.995549000000 0.895815000000 2.182347000000

Cu -1.052144000000 1.835710000000 -2.431642000000

Cu -2.385389000000 -2.632250000000 -1.172838000000

Cu 1.667604000000 3.795290000000 0.938410000000

Cu -2.071166000000 -0.433489000000 -2.431532000000

38

6.768842500 0008.out

Cu 3.378495000000 -1.097740000000 -1.172970000000

Cu 0.000000000000 2.145197000000 0.032772000000

Cu 2.012643000000 0.653947000000 -2.431766000000

Cu -3.334103000000 2.463807000000 0.938557000000

Cu 0.000000000000 0.000000000000 1.123689000000

Cu -2.088025000000 2.873919000000 -1.172970000000

Cu -3.378495000000 -1.097740000000 -1.172970000000

Cu -3.373514000000 -2.409562000000 0.938557000000

Cu 2.040204000000 0.662902000000 0.032772000000

Cu 1.260915000000 -1.735501000000 0.032772000000

Cu 1.243882000000 -1.712056000000 -2.431766000000

Cu 4.145535000000 -0.033525000000 0.938557000000

Cu 2.079873000000 -0.675792000000 2.182292000000

Cu 1.249157000000 -3.952998000000 0.938557000000

Cu -1.249157000000 -3.952998000000 0.938557000000

Cu 4.026559000000 1.308308000000 -1.146004000000

Cu 1.285432000000 1.769246000000 2.182292000000

Cu 0.000000000000 4.233775000000 -1.146004000000

Cu 2.488551000000 -3.425196000000 -1.146004000000

Cu 2.088025000000 2.873919000000 -1.172970000000

Cu 0.000000000000 -2.186908000000 2.182292000000

Cu 0.000000000000 0.000000000000 3.439811000000

Cu 1.312925000000 3.932278000000 0.938557000000

Cu 0.000000000000 2.116218000000 -2.431766000000

Cu -2.488551000000 -3.425196000000 -1.146004000000

Cu -1.260915000000 -1.735501000000 0.032772000000

Cu 3.373514000000 -2.409562000000 0.938557000000

Cu 0.000000000000 0.000000000000 -1.270691000000

Cu -4.026559000000 1.308308000000 -1.146004000000

Cu -1.285432000000 1.769246000000 2.182292000000

Cu -4.145535000000 -0.033525000000 0.938557000000

Cu -2.040204000000 0.662902000000 0.032772000000

Cu 3.334103000000 2.463807000000 0.938557000000

Cu -2.079873000000 -0.675792000000 2.182292000000

Cu -2.012643000000 0.653947000000 -2.431766000000

Cu 0.000000000000 -3.552360000000 -1.172970000000

Cu -1.312925000000 3.932278000000 0.938557000000

Cu -1.243882000000 -1.712056000000 -2.431766000000

38

6.771352500 0009.out

Cu 3.378495000000 -1.097740000000 -1.172928000000

Cu 0.000000000000 2.145197000000 0.032814000000

Cu 2.012643000000 0.653947000000 -2.431724000000

Cu -3.334082000000 2.463861000000 0.938439000000

Cu 0.000000000000 0.000000000000 1.123731000000

Cu -2.088025000000 2.873919000000 -1.172928000000

Cu -3.378495000000 -1.097740000000 -1.172928000000

Cu -3.373559000000 -2.409526000000 0.938439000000

Cu 2.040204000000 0.662902000000 0.032814000000

Cu 1.260915000000 -1.735501000000 0.032814000000

Cu 1.243882000000 -1.712056000000 -2.431724000000

Cu 4.145550000000 -0.033581000000 0.938439000000

Cu 2.079873000000 -0.675792000000 2.182334000000

Cu 1.249108000000 -3.953030000000 0.938439000000

Cu -1.249108000000 -3.953030000000 0.938439000000

Cu 4.026559000000 1.308308000000 -1.145962000000

Cu 1.285432000000 1.769246000000 2.182334000000

Cu 0.000000000000 4.233775000000 -1.145962000000

Cu 2.488551000000 -3.425196000000 -1.145962000000

Cu 2.088025000000 2.873919000000 -1.172928000000

Cu 0.000000000000 -2.186908000000 2.182334000000

Cu 0.000000000000 0.000000000000 3.439853000000

Cu 1.312983000000 3.932276000000 0.938439000000

Cu 0.000000000000 2.116218000000 -2.431724000000

Cu -2.488551000000 -3.425196000000 -1.145962000000

Cu -1.260915000000 -1.735501000000 0.032814000000

Cu 3.373559000000 -2.409526000000 0.938439000000

Cu 0.000000000000 0.000000000000 -1.270649000000

Cu -4.026559000000 1.308308000000 -1.145962000000

Cu -1.285432000000 1.769246000000 2.182334000000

Cu -4.145550000000 -0.033581000000 0.938439000000

Cu -2.040204000000 0.662902000000 0.032814000000

Cu 3.334082000000 2.463861000000 0.938439000000

Cu -2.079873000000 -0.675792000000 2.182334000000

Cu -2.012643000000 0.653947000000 -2.431724000000

Cu 0.000000000000 -3.552360000000 -1.172928000000

Cu -1.312983000000 3.932276000000 0.938439000000

Cu -1.243882000000 -1.712056000000 -2.431724000000
